# Clustering of hypertension and clustering of diabetes within households across districts of India: A cross-sectional analysis using a nationally representative household survey

**DOI:** 10.1371/journal.pgph.0004648

**Published:** 2025-06-17

**Authors:** Sarang Pradipkumar Pedgaonkar, Kaushalendra Kumar, Wahengbam Bigyananda Meitei, Shubham Kumar, Ashish Kumar Upadhyay, Jürgen Maurer, Abhishek Singh

**Affiliations:** 1 Department of Family and Generations, International Institute for Population Sciences, Mumbai, Maharashtra, India; 2 Department of Public Health and Mortality Studies, International Institute for Population Sciences, Mumbai, Maharashtra, India; 3 GENDER Project, International Institute for Population Sciences, Mumbai, Maharashtra, India; 4 NFHS-6 Project, International Institute for Population Sciences, Mumbai, Maharashtra, India; 5 Department of Economics, Faculty of Business and Economics (HEC), University of Lausanne and Lausanne Center for Health Economics, Behavior and Policy (LCHE), Lausanne, Switzerland; 6 Centre of Demography of Gender, International Institute for Population Sciences, Mumbai, Maharashtra, India; Indian Institute of Public Health Hyderabad, INDIA

## Abstract

Despite the rising prevalence of hypertension and diabetes, limited evidence exists on clustering of hypertension and clustering of diabetes within households in India, prompting this study to examine the issue among individuals aged 15 years and above across all 707 districts in India. Clustering here is defined as two or more household members having a disease. We examined clustering for hypertension and clustering for diabetes separately, using cross-sectional data from 5^th^ round of India DHS (National Family Health Survey-5, 2019–21). The factors influencing clustering at the community, district, and state levels were evaluated by multi-level analysis. In India, 14.9% of households had hypertension clustering, contributing to half of the total cases of hypertension in India, while 7.7% had diabetes clustering, accounting for 39.3% of total cases of diabetes in India. Distinct concentrated regions with high prevalence of clustering were noted across districts in India. The regressions at national level revealed that clustering for both diseases was more likely in large households with higher number of older members, wealthier households, households with an overweight woman, regular consumption of fish and fried food, and urban residence. The ICC for clustering was highest at the community level, highlighting the highest impact of factors in the immediate neighbourhood. Clustering within households is evident for both hypertension and diabetes. By providing quantitative estimates of disproportionate case burden among clustered households, our findings underscore the importance of targeting households for effective hypertension and diabetes management interventions. These results provide valuable insights about district-wise distribution of hypertension and diabetes within the unique context of household level clustering, equipping health systems with information on concentrated disease burden and key driving factors influencing clustering in India. This may inform intensified interventions, accelerating progress towards SDG 3·4.

## Introduction

Non-communicable diseases (NCDs) in India pose a serious public health challenge both in terms of high and increasing prevalence and low levels of awareness, diagnosis and adequate treatment [1–3]. In India, age-standardized prevalence of hypertension has been estimated to increase from 24.5% to 26.6% among men and from 22.7% to 24.7% among women between 1980 and 2014. Similarly, the prevalence of diabetes has been estimated to rise from 3.7% to 9.1% among men and from 4.6% to 8.3% among women during the same period [4]. As per recent estimates, approximately 220 million people are suffering from hypertension and 77 million people are suffering from diabetes in India [5–7]. However, only 54% of people with hypertension and 46% of people with diabetes are aware about their disease status and just half of the people with hypertension and 36% of the people with diabetes are under medication [8,9]. In addition, there are wide socioeconomic and geographic variations in the distribution of these diseases in India [4,10].

Specific NCDs are often more concentrated within families due to shared genetic factors, common environmental exposures and social transmission of lifestyle, nutrition patterns, and other high-risk behaviours associated with NCDs [11,12]. The term familial clustering, which refers to concentration of certain phenomenon within families, is frequently used in mortality research [13,14]. Here we are using the term clustering in the context of NCDs to denote concentration of NCDs within certain families, and not for statistical grouping. Familial clustering of NCDs is well documented, mostly in studies from western countries [15–20]. Though limited, existing evidence from India also suggests familial clustering of risk factors for NCDs including hypertension and diabetes [21–25]. Attention to such household level clustering is essential for complete understanding of the epidemiology of hypertension and diabetes in India. Along with providing valuable insights about cumulative health disadvantage across households, examination of clustering will help in better understanding of the complex interactions between genetic predispositions, environmental factors, and social influences that may result in disease clustering within households. Additionally, these clustered households are at disproportionately higher risk to experience the complications of these diseases in the form of cardiovascular issues like heart attack and stroke, putting these households in excessive need of healthcare and other supports. Unless addressed by effective interventions, eventually this may escalate to clustering of mortality due to these complications within household with clustering.

The high-risk behaviours linked to modifiable risk factors for NCDs are shaped by socio-cultural and economic influences and are liable to be transmitted from person to person as per human interactions [26–29]. In addition to shared risks and the social transmission of high-risk behaviours and chronic conditions within families, the immediate support may also arise within the families. These same social ties can be leveraged to foster support, promote healthy behaviour changes, and improve disease management through better awareness, treatment adherence and complication prevention [30–32]. Therefore, proper understanding of phenomenon of clustering will be immensely helpful to potentially strategize interventions for prevention, diagnosis, and management of hypertension and diabetes to be targeted at the household level in addition to the interventions targeted at the individual level.

India is one of the most populous and diverse countries with large geo-climatic, cultural, dietary, sociodemographic, and economic differences. In terms of burden of hypertension and diabetes, different states in India have widely different levels of disease [1,3,9]. States in India are also not homogenous units and different regions within a state vary considerably in terms of climate, urbanization, and economic activities [33–36]. Therefore, to better understand spatial patterns of NCD epidemiology in India, one must look in more detail beyond the state level. Districts in India are administrative units and also the units for the health-related planning and execution. Examination of household level clustering of hypertension and diabetes at the district level is, therefore, especially helpful for the efficient and inclusive planning and implementation of interventions for diagnosis, prevention, and management of these diseases in line with the priorities of the districts.

There are limited studies examining familial clustering of hypertension or diabetes in India. These existing studies are mainly hospital based and only consider selected geographical areas or population groups [37–42]. Only a few studies have attempted to examine familial clustering of chronic conditions and hypertension using representative samples in India. However, the focus of these studies is typically limited to considerations of concordance of hypertension within married couples [21–23,43]. The present study delves into the phenomenon of clustering of hypertension and diabetes within households more broadly and examines the distribution and determinants of clustering across all 707 districts in India.

The existing literature highlighted the role of neighbourhood factors like health relevant environments, behavioural practices, social factors, and health policies in explaining health or disease status of an individual independent of other individual-level characteristics [44,45]. Households share common exposure to various neighbourhood factors along with common environment exposures which are modulated at different levels of population settlements and administration units like community, district and state. Examination of clustering of these diseases among household members using hierarchical data and applying multilevel modelling can provide insights into the role played by such factors applicable at different levels like community, district, and state. The role of neighbourhood factors and variations at different population levels in the context of NCDs has not received much attention in India. Very few studies from India have examined such variations. However, they have focused on overall prevalence of NCDs, risk factors for NCDs, and limited to particular states in India [25,46,47]. This study examined the variation in clustering of hypertension and diabetes within households across India attributable to various factors at community, district, and state level.

## Methods

### Data source

We used data from the fifth round of the National Family Health Survey (NFHS-5) conducted in India during June, 2019 to April, 2021. NFHS-5, also known as India Demographic and Health Survey, is a nationally representative household survey conducted across 28 states and 8 union territories of India. NFHS-5 used a two-stage stratified sampling design in both urban and rural areas. The detailed information on sampling design can be found elsewhere [48]. The total number of households interviewed in NFHS-5 were 636699, with a response rate of 98%. Of these, 8 households comprised only of transgender members, which were not considered for further analysis due to small number.

A total of 619839 households were considered for analysis for clustering of hypertension where blood pressure (BP) measurement was completed for at-least one member of the household. Information about consumption of different food items was collected by interviewing women age 15–49 years and weight and height measurements were taken after completion of interview. Therefore, analysis involving consumption of food items and body mass index (BMI) was restricted to 500267 households where at-least one woman’s interview was completed along with measurement of blood pressure of at least one member of the household.

Similarly, clustering for diabetes was examined in 615125 households where random blood glucose (RBG) testing was completed for at-least one member of the household and further analysis involving consumption of food items and BMI was restricted to 497949 households where any eligible woman’s interview was completed along with RBG measurement. We followed the Strengthening the Reporting of Observational Studies in Epidemiology (STROBE) guideline in reporting the study (S1 Checklist).

### Measurement

In NFHS-5, BP and RBG level of eligible men and women age 15 years or above in the surveyed households were measured using standard equipment and following standardized protocol. These measurements were done by paramedical personnel who were rigorously trained for two weeks and followed by field practice of at-least 3 days. The detailed protocol for measurement of anthropometry, BP and blood glucose can be found elsewhere [49]. BP was measured after the completion of survey questionnaire from eligible respondents and after obtaining informed consent. BP was measured in a single home visit using Omron HEM 812 automatic digital BP monitor manufactured by Omron Healthcare Vietnam Co. Ltd, Vietnam. A total of three measurements were taken, preferably on the left arm in a sitting position, with a five-minute gap between two consecutive measurements. The average systolic and diastolic blood pressure from second and third measurements were considered as the final measurement for analysis. Random blood glucose was measured using Accu-Chek Performa glucometer manufactured by Roche Diabetes Care, Inc. USA by drawing capillary blood after obtaining informed consent. Along with measurement, all the eligible respondents who consented for blood pressure measurement were asked questions about diagnosis prior to survey- *were you told on two or more occasions by a doctor, nurse or auxiliary nurse midwife (ANM) that you had hypertension or high blood pressure?* and *to lower your blood pressure, are you now taking a prescribed medicine?* Similarly, all the eligible respondents who consented for blood glucose measurement were asked questions about diagnosis prior to survey- *were you told on two or more occasions by a doctor, nurse or auxiliary nurse midwife (ANM) that your blood glucose level was high?* and *to lower your blood glucose level, are you now taking a prescribed medicine?*

Among the respondents, hypertensive were identified as those having Systolic Blood Pressure (SBP) ≥ 140 mmHg or Diastolic Blood Pressure (DBP) ≥ 90 mmHg or taking any medication to lower blood pressure at the time of survey (S2 Text). Likewise, diabetics were identified as those having a random blood glucose level greater than 140 mg/dL or taking any medicines for diabetes (S2 Text).

The details of weight and height measurement can be found in S1 Text. The detailed protocol of biomarkers and biomarker questionnaire can be found elsewhere [49,50].

### Analytical procedure

Household-level clustering for a disease occurs when a household has two or more members identified with that particular disease. So, household-level clustering for hypertension occurs when two or more members of a household are identified with hypertension (S2 Text). Similarly, household-level clustering for diabetes occurs when two or more members of a household are identified with diabetes (S2 Text). We analysed the clustering for hypertension and diabetes separately.

Variations in clustering at the household level were assessed by various characteristics of the household, head of the household, and the PSU. The household characteristics included in the analysis are number of members age 15–30 years, 31–50 years, 51–60 years, 61–70 years and 71 years and above in the household, share of 15 + years women in 15 + years members of the household, number of members of the household drinking alcohol, number of members consuming tobacco in any form, household’s economic status (wealth quintiles) and presence of any overweight/obese woman in the household. When it comes to the characteristics of the household head, caste, religion, and education of the head of household were included in the analysis. The PSU level characteristics included for analysing clustering were place of residence of the household and percentage of *pucca* households within the PSU. The percentage of *pucca* households in the PSU was taken as a proxy indicator of economic status of the immediate surroundings.

In the absence of information about overweight/obesity status for all household members in NFHS-5, we included the presence of any overweight/obese woman in the household as a proxy indicator for the presence of overweightness/obesity in the household. A wealth index is considered a good proxy indicator of the economic status of the households in NFHS where income or expenditure data is not collected [51]. The wealth index was constructed by principal component analysis using data on household’s ownership of selected assets, access to utilities and infrastructure, and housing characteristics of households surveyed in NFHS-5 [48].

In NFHS-5 men were not interviewed in all of the communities (PSUs). In the absence of information from interviews of men in all PSUs, the information of different types of food consumption collected by interviewing women age 15–49 years in all households is considered as a proxy indicator for food consumption by entire household, which should be reasonably reliable as food preparation is usually same for the entire household. Also, in the households where both men and women were interviewed, the consumption of almost all food items considered in our analysis was higher among men than women [48,52]. The food items included in the analysis are daily or weekly consumption of milk or milk products, chicken or meat, fried food, and aerated drinks.

We estimated four-level random intercept logistic regressions to examine the association of household-, community-, district- and state- level variables with household clustering of hypertension and diabetes. We also estimated the intraclass correlation coefficient (ICC) at community, district, and state levels.

A four-level random intercept logistic regression model can be mathematically represented as


$$Y_{ijkl}=\log(\frac{\pi_{ijk}}{1-\pi_{ijkl}})=a+\beta X_{ijkl}+\gamma Z_{jkl}+\delta W_{kl}+\phi U_{l}+r_{0l}+s_{0kl}+d_{0jkl}+e_{0ijkl}$$

where $Y_{ijkl}$ is the household with clustering of selected NCD for household *i* in the community *j* in district *k* in state *l*. a is constant, $X_{ijkl},$ $Z_{jkl},\;W_{kl},\;and\;U_{l}$ are the vectors of the variables. β, γ, δ, and ϕ are the regression coefficients. $r_{0l},\;s_{0kl},\;d_{0jkl},\;and\;e_{0ijkl}\;$ are the residua*l*s at household, community, district, and state levels, respectively.


$$ICC=\frac{VAR_{n}}{\{\sum_{n=2}^{N}VAR_{n}+\left(\frac{\pi^{2}}{3}\right\}}$$

where ICC is the intraclass correlation coefficient and $VAR_{n}$ is the variance at the n^th^ level of regression.

Four-level random intercept logistic regressions were estimated on two sets of samples. First set of samples consisted of households where blood pressure and blood glucose were measured in the households. As food consumption and BMI were available only for women age 15–49, we estimated our models on a second set of samples. The second set of samples is a sub-set of the first set and consisted of households where women age 15–49 were interviewed along with blood pressure and blood glucose measurements. The variables added in the second set of analyses were daily or weekly consumption of milk or milk products, chicken or meat, fried food, and aerated drinks and presence of any overweight/obese woman in the household. Since men were not interviewed in all of the communities (PSUs), information from men’s interview or anthropometry measurements were not included in the regression models.

In all our analyses, we incorporated sampling weights and accounted for both stratification and cluster sampling to estimate confidence intervals (CIs). The analysis was done using MLwiN, STATA 16, and ArcGIS [53–55].

### Ethical consideration

Our study uses secondary data from NFHS-5 which is available for public use. NFHS-5 obtained ethical approval from the Institutional Review Boards at the International Institute for Population Sciences (IIPS) and its collaborating institutions. The NFHS-5 data used in this study are publicly available at https://www.iipsdata.ac.in/datacatalog_detail/1. The NFHS-5 dataset do not have any information that could identify respondents’ identities, households, or sample communities. So ethical approval is not required for our study.

## Results

**Fig 1:**
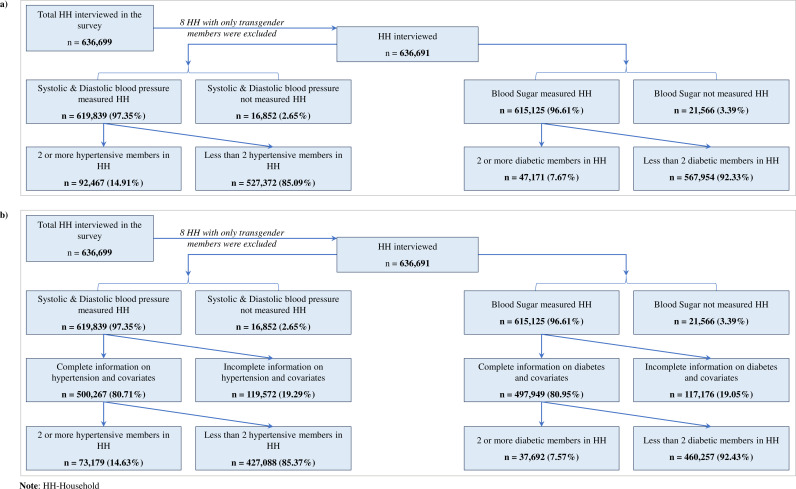
Flowchart of household selection: (a) full sample, and (b) sub-sample households where interview of any eligible woman, 15-49 years was completed.

Fig 1 shows the selection of analytical sample. Blood pressure could not be measured in any member in 16852 households because participants did not give consent, were not available for measurement, or had open sore or wound or irritations on both hands that prevented fitting of cuff of BP monitor. Similarly, RBG could not be measured in any member in 21566 households because participants did not give consent or were not available for measurement. Out of the 619839 households where BP was measured, in 119572 households the interview and height and weight measurement of any woman aged 15–49 years could not be done because no age eligible woman was member of the household, eligible woman did not give consent, or was not available for interview. Similarly, among the 615125 households with RBG measurement, in 117176 households the interview and height and weight measurement of any woman aged 15–49 years could not be done because no age eligible woman was member of the household, eligible woman did not give consent, or was not available (S1 and S2 Tables). Table 1 shows the characteristics of the surveyed households. The characteristics of the households analysed for clustering of hypertension were similar to those analysed for clustering of diabetes. The mean number of household members age 15–30 years was 1.3, those age 31–50 years was 1.1, age 51–60 years was 0.5, age 61–70 years was 0.3, and for that those age 71 and above was 0.1. On average, women made up slightly more than half of the household members age 15 and older. No member age 15 years and above consumed alcohol in 73.1% of households, only one member consumed alcohol in 22.5% households, two members consumed alcohol in 3.5% of households, whereas in only one percent households three or more members consumed alcohol. No member consumed tobacco in almost half (46.6%) of the households, only one member used tobacco in 36.6% of households, two members used tobacco in 12.3% of households, and three or more members consumed tobacco in 4.5% of households. About four-tenths (41.7%) of household heads belonged to other backward classes (OBC), 21.8% belonged to scheduled castes (SC), 9.7% belonged to scheduled tribes (ST), and 26.8% belonged to other castes. Majority of the household heads belonged to Hindu religion (82.2%), followed by Muslim (12.2%), Christian (2.8%), and Sikh (1.5%). Forty-two percent household heads had completed secondary schooling and 10.5% had completed higher than secondary schooling. About a-fifth (18.8%) of the household heads completed primary schooling and 28.7% did not receive any schooling. About two-thirds (67.9%) of the households resided in rural areas. On an average, proportion of *pucca* houses in the community was 59.7%. Based upon women’s interview, milk or milk products were consumed on daily or weekly basis by 75.2% women, fish and meat by 40.0% and 40.1% respectively, fried food by 47.3%, and aerated drinks by 18.0%. At least one woman was either overweight or obese in 30.4% of households where women were interviewed and BP or RBG were measured.

**Table 1:** Sample characteristics.

	Households analysed for Hypertension				Households analysed for Diabetes			
	%	95% CI	% @	95% CI @	%	95% CI	% @	95% CI @
Number of 15–30 years members in the HH (mean)	1.3	[1.3, 1.3]	1.5	[1.5, 1.5]	1.3	[1.3, 1.3]	1.5	[1.5, 1.5]
Number of 31–50 years members in the HH (mean)	1.1	[1.1, 1.1]	1.3	[1.3, 1.3]	1.1	[1.1, 1.1]	1.3	[1.3, 1.3]
Number of 51–60 years members in the HH (mean)	0.5	[0.5, 0.5]	0.4	[0.4, 0.4]	0.5	[0.5, 0.5]	0.4	[0.4, 0.4]
Number of 61–70 years members in the HH (mean)	0.3	[0.3, 0.3]	0.2	[0.2, 0.2]	0.3	[0.3, 0.3]	0.2	[0.2, 0.2]
Number of 71 + years members in the HH (mean)	0.1	[0.1, 0.1]	0.1	[0.1, 0.1]	0.1	[0.1, 0.1]	0.1	[0.1, 0.1]
% Share of 15 + years female members in the HH (mean)	52.8	[52.8, 52.9]	54.3	[54.2, 54.3]	52.8	[52.7, 52.8]	54.3	[54.2, 54.3]
No. of HH member drink alcohol								
No member	73.1	[73, 73.2]	72.1	[72.0, 72.2]	73.0	[72.9, 73.1]	72.0	[71.9, 72.1]
One member	22.5	[22.4, 22.7]	23.3	[23.2, 23.4]	22.6	[22.5, 22.7]	23.4	[23.2, 23.5]
Two members	3.5	[3.4, 3.5]	3.7	[3.6, 3.7]	3.5	[3.4, 3.5]	3.7	[3.6, 3.7]
Three and more members	0.9	[0.8, 0.9]	0.9	[0.9, 1.0]	0.9	[0.8, 0.9]	0.9	[0.9, 1.0]
No. of HH member smoke and/or use tobacco								
No member	46.6	[46.5, 46.7]	45.3	[45.2, 45.5]	46.5	[46.4, 46.6]	45.3	[45.1, 45.4]
One member	36.6	[36.4, 36.7]	36.8	[36.7, 37.0]	36.6	[36.5, 36.7]	36.9	[36.7, 37.0]
Two members	12.3	[12.2, 12.4]	12.8	[12.7, 12.9]	12.4	[12.3, 12.4]	12.8	[12.7, 12.9]
Three and more members	4.5	[4.5, 4.6]	5.1	[5.0, 5.1]	4.5	[4.5, 4.6]	5.1	[5.0, 5.1]
HH head’s education								
No education or below primary	28.7	[28.6, 28.8]	27.0	[26.9, 27.1]	28.6	[28.5, 28.7]	27.0	[26.9, 27.1]
Primary completed	18.8	[18.7, 18.9]	18.6	[18.5, 18.8]	18.8	[18.7, 18.9]	18.7	[18.6, 18.8]
Secondary completed	42.0	[41.9, 42.1]	44.1	[44.0, 44.2]	42.1	[41.9, 42.2]	44.1	[44.0, 44.2]
Higher secondary and above	10.5	[10.4, 10.6]	10.3	[10.2, 10.3]	10.5	[10.4, 10.6]	10.2	[10.1, 10.3]
HH Wealth Quintile								
Lowest	21.1	[21.0, 21.2]	19.9	[19.8, 20]	21.1	[21.0, 21.2]	19.9	[19.8, 20.1]
Second	20.3	[20.2, 20.4]	20.4	[20.3, 20.5]	20.3	[20.2, 20.5]	20.4	[20.3, 20.5]
Middle	20.2	[20.1, 20.3]	20.6	[20.5, 20.7]	20.3	[20.2, 20.4]	20.6	[20.5, 20.7]
Higher	19.6	[19.5, 19.7]	20.3	[20.2, 20.4]	19.6	[19.5, 19.7]	20.3	[20.2, 20.4]
Highest	18.7	[18.6, 18.8]	18.8	[18.7, 18.9]	18.6	[18.5, 18.7]	18.7	[18.6, 18.9]
Social group of the HH head								
Scheduled Tribe	9.7	[9.6, 9.7]	9.9	[9.8, 10.0]	9.7	[9.6, 9.8]	9.9	[9.9, 10.0]
Scheduled Caste	21.8	[21.7, 21.9]	22.3	[22.2, 22.4]	21.9	[21.8, 22.0]	22.3	[22.2, 22.5]
Other Backward Class	41.7	[41.5, 41.8]	41.5	[41.4, 41.7]	41.6	[41.5, 41.8]	41.5	[41.4, 41.7]
Others	26.8	[26.7, 26.9]	26.2	[26.1, 26.3]	26.8	[26.7, 26.9]	26.2	[26.1, 26.3]
Religion of the HH head								
Other religion	1.3	[1.3, 1.3]	1.3	[1.2, 1.3]	1.3	[1.3, 1.3]	1.3	[1.2, 1.3]
Hindu	82.2	[82.1, 82.3]	81.6	[81.5, 81.7]	82.3	[82.2, 82.4]	81.7	[81.6, 81.8]
Muslim	12.2	[12.1, 12.3]	13.1	[13.0, 13.2]	12.1	[12.0, 12.2]	13.0	[12.9, 13.1]
Christian	2.8	[2.8, 2.9]	2.6	[2.5, 2.6]	2.8	[2.8, 2.8]	2.6	[2.5, 2.6]
Sikh	1.5	[1.4, 1.5]	1.5	[1.4, 1.5]	1.4	[1.4, 1.5]	1.5	[1.4, 1.5]
Place of residence								
Urban	32.1	[32.0, 32.2]	31.6	[31.4, 31.7]	32.0	[31.9, 32.2]	31.5	[31.4, 31.6]
Rural	67.9	[67.8, 68.0]	68.4	[68.3, 68.6]	68.0	[67.8, 68.1]	68.5	[68.4, 68.6]
Proportion of pucca HH in a PSU (mean)	0.6	[0.6, 0.6]	0.6	[0.6, 0.6]	0.6	[0.6, 0.6]	0.6	[0.6, 0.6]
HH consume milk or milk product daily or weekly								
No								
Yes			75.2	[75.1, 75.3]			75.2	[75.1, 75.3]
HH consume fish daily or weekly								
No								
Yes			40.0	[39.9, 40.2]			40.0	[39.9, 40.2]
HH consume chicken or meat daily or weekly								
No								
Yes			40.1	[40.0, 40.2]			40.1	[39.9, 40.2]
HH consume fried foods daily or weekly								
No								
Yes			47.3	[47.1, 47.4]			47.3	[47.1, 47.4]
HH consume aerated drinks daily or weekly								
No								
Yes			18.0	[17.9, 18.1]			18.0	[17.9, 18.1]
Presence of overweight/obese women in the HH								
No								
Yes			30.4	[30.3, 30.6]			30.4	[30.3, 30.5]

**Note**: HH-household; PSU-Primary Sampling Unit; ^@^ in the sub-sample households where interview of any eligible woman (15–49 years) was completed.

Table 2 shows the percent distribution of households (HHs) by number of members identified with hypertension and diabetes, respectively, along with the proportion of total cases nested within them. Among 14.9% households two or more members age 15 years or above were identified with hypertension. Only two members age 15 years or above were identified with hypertension in 12.0% of households, three members in 2.4% households and four, five, and six or more members were identified in less than one percent of households each. About half of the total case burden of hypertension in India was nested within these 14.9% of households with clustering. Only one member was identified with hypertension in 33.7% of households, harbouring half of the case burden of hypertension. In 51.4% of the households, not a single member was identified with hypertension.

**Table 2:** Percentage distribution of households with number of members identified with hypertension and diabetes along with the proportion of total cases nested within the household.

No. of HH members identified with hypertension	Number of HH for hypertension	Percentage of total HH	Proportion of total case burden of hypertension	Number of HH for hypertension^@^	Percentage of total HH^@^	Proportion of total case burden of hypertension^@^	Number of HH for diabetes	Percentage of total HH	Proportion of total case burden of diabetes	Number of HH for diabetes^@^	Percentage of total HH^@^	Proportion of total case burden of diabetes^@^
0	318,683	51.4	0	267,549	53.5	0.00	409,152	66.5	0	337,592	67.8	0.00
1	208,690	33.7	50.2	159,539	31.9	49.0	158,803	25.8	60.7	122,666	24.6	59.6
2	74,440	12.0	35.8	56,932	11.4	35.0	40,078	6.5	30.7	31,181	6.7	30.3
3	14,659	2.7	10.6	13,031	2.6	12.0	5,986	1.0	6.8	5,435	1.1	7.9
4	2,718	0.4	2.6	2,576	0.5	3.1	927	0.2	1.4	900	0.2	1.7
5	520	0.1	0.6	512	0.1	0.8	138	0.0	0.2	136	0.0	0.4
6+	130	0.0	0.2	128	0.	0.2	42	0.0	0.1	40	0.0	0.2
2 or more	92,467	14.9	49.8	73,179	14.6	51.0	47,171	7.7	39.3	37,692	7.6	40.4
**Total**	**619,839**			**500,267**			**615,125**			**497,949**		

**Note**: HH-Household; ^@^ in the sub-sample households where interview of any eligible woman (15–49 years) was completed.

In one-twelfth of households (7.7%) two or more members age 15 years or above were identified with diabetes, which contributed to 39.3% of total case burden of diabetes in India. Only two members age 15 and above were identified with diabetes in around 6.8% of the households, three members in around 1.4% of the households whereas four, five, and six or more members were identified with diabetes in less than 1% of the households each. In 25.8% of households only one member was identified with diabetes, bearing 60.7% of total case load of diabetes whereas, in 66.5% of households no member was identified with diabetes.

Table 3 shows the prevalence of clustering of hypertension and diabetes by selected household characteristics. The prevalence of clustering of hypertension was higher among urban households (17.1% than rural households (13.9%). Among the caste groups, the prevalence of clustering of hypertension was highest among households headed by other castes (17.1%). The prevalence of clustering of hypertension was lowest among households headed by ST (12.9%). Among the religious groups, Sikhs had the highest prevalence of clustering of hypertension (29.4%), followed by Christians (17.3%), Hindus (14.8%), and Muslims (13.6%). Among lowest wealth quintile households, clustering of hypertension was observed in 8.8% households, which increased to 22.2% in highest wealth quintile households. The prevalence of clustering increased with the number of members per household consuming alcohol or tobacco. The prevalence of clustering of hypertension increased from 14.3% among households with only one member consumed alcohol to 26.4% where three or more members consumed alcohol. The prevalence of clustering for hypertension increased from 14.9% among households with only one member using tobacco to 23% among households with three or more members using tobacco. The prevalence of clustering for hypertension was more in households that consumed milk or milk products (15.4%), fried food (15.2%), and aerated drinks (15.9%) daily or weekly.

**Table 3:** Prevalence of two or more member in the household suffering from hypertension or diabetes by selected household characteristics.

	2 or more hypertensive members in the HH				2 or more diabetic members in the HH			
	%	95% CI	% ^@^	95% CI ^@^	%	95% CI	% ^@^	95% CI ^@^
No. of HH member drink alcohol								
No member	14.6	[14.5, 14.8]	14.3	[14.2, 14.5]	7.9	[7.8, 8.0]	7.8	[7.6, 7.9]
One member	14.3	[14.1, 14.6]	13.9	[13.7, 14.2]	6.6	[6.5, 6.8]	6.5	[6.3, 6.7]
Two members	21.7	[21.1, 22.4]	21.8	[21.0, 22.5]	8.8	[8.3, 9.2]	9.1	[8.6, 9.7]
Three and more members	26.4	[25.1, 27.8]	27.3	[25.8, 28.7]	11.1	[10.1, 12.3]	11.3	[10.2, 12.4]
No. of HH member smoke and/or use tobacco								
No member	14.9	[14.7, 15.0]	14.4	[14.2, 14.6]	8.0	[7.8, 8.1]	7.7	[7.5, 7.8]
One member	13.1	[13.0, 13.3]	12.9	[12.7, 13.1]	6.5	[6.4, 6.7]	6.5	[6.4, 6.7]
Two members	17.4	[17.1, 17.8]	17.1	[16.8, 17.5]	8.4	[8.1, 8.7]	8.5	[8.2, 8.9]
Three and more members	23.0	[22.4, 23.6]	23.2	[22.6, 23.9]	11.5	[11.0, 12.0]	11.9	[11.4, 12.5]
HH head’s education								
No education or below primary	12.8	[12.6, 13.0]	13.2	[13.0, 13.4]	5.9	[5.7, 6.1]	6.2	[6.0, 6.4]
Primary completed	15.3	[15.0, 15.6]	14.8	[14.5, 15.1]	7.8	[7.6, 8.0]	7.7	[7.5, 8.0]
Secondary completed	15.7	[15.5, 15.9]	15.0	[14.8, 15.2]	8.3	[8.2, 8.5]	8.0	[7.8, 8.1]
Higher secondary and above	17.0	[16.5, 17.4]	16.4	[15.9, 16.9]	9.6	[9.3, 10.0]	9.0	[8.6, 9.4]
HH Wealth Quintile								
Lowest	8.8	[8.6, 9.0]	8.2	[8.0, 8.5]	4.2	[4.0, 4.3]	4.2	[4.1, 4.4]
Second	11.7	[11.5, 11.9]	11.4	[11.1, 11.6]	5.5	[5.4, 5.7]	5.6	[5.4, 5.8]
Middle	14.7	[14.4, 15.0]	14.5	[14.2, 14.8]	7.4	[7.2, 7.6]	7.3	[7.1, 7.5]
Higher	18.1	[17.8, 18.4]	17.7	[17.3, 18.0]	9.7	[9.4, 9.9]	9.4	[9.1, 9.7]
Highest	22.2	[21.9, 22.6]	21.8	[21.4, 22.2]	12.2	[11.9, 12.5]	11.6	[11.3, 12.0]
Social group of the HH head								
Scheduled Tribes	12.9	[12.6, 13.2]	12.7	[12.4, 13.1]	4.9	[4.7, 5.1]	5.0	[4.7, 5.2]
Scheduled Castes	13.2	[12.9, 13.4]	12.9	[12.6, 13.2]	6.3	[6.1, 6.5]	6.3	[6.0, 6.5]
Other Backward Class	14.9	[14.7, 15.1]	14.7	[14.5, 14.9]	7.8	[7.7, 8.0]	7.7	[7.6, 7.9]
Others	17.1	[16.9, 17.4]	16.7	[16.4, 17.0]	9.6	[9.3, 9.8]	9.4	[9.2, 9.7]
Religion of the HH head								
Other religion	15.2	[14.1, 16.4]	15.1	[13.8, 16.4]	5.6	[4.9, 6.5]	5.7	[4.8, 6.7]
Hindu	14.8	[14.6, 14.9]	14.5	[14.4, 14.7]	7.6	[7.5, 7.7]	7.5	[7.3, 7.6]
Muslim	13.6	[13.3, 14.0]	13.3	[12.9, 13.6]	7.9	[7.6, 8.2]	8.0	[7.7, 8.3]
Christian	17.3	[16.5, 18.1]	16.1	[15.2, 17.0]	10.1	[9.5, 10.8]	9.5	[8.8, 10.2]
Sikh	29.4	[28.4, 30.3]	30.0	[28.9, 31.0]	8.0	[7.4, 8.5]	7.9	[7.3, 8.5]
Place of residence								
Urban	17.1	[16.8, 17.4]	16.7	[16.4, 17.0]	9.5	[9.3, 9.7]	9.2	[9.0, 9.5]
Rural	13.9	[13.8, 14.0]	13.7	[13.5, 13.8]	6.8	[6.7, 6.9]	6.8	[6.7, 6.9]
HH consume milk or milk product daily or weekly								
No			12.1	[11.9, 12.4]			5.9	[5.7, 6.1]
Yes			15.4	[15.3, 15.6]			8.1	[8.0, 8.3]
HH consume fish daily or weekly								
No			14.6	[14.5, 14.8]			6.5	[6.4, 6.7]
Yes			14.6	[14.4, 14.8]			9.1	[8.9, 9.3]
HH consume chicken or meat daily or weekly								
No			14.5	[14.3, 14.6]			6.8	[6.7, 7.0]
Yes			14.9	[14.6, 15.1]			8.7	[8.5, 8.9]
HH consume fried foods daily or weekly								
No			14.1	[13.9, 14.3]			6.9	[6.7, 7.0]
Yes			15.2	[15.0, 15.4]			8.3	[8.2, 8.5]
HH consume aerated drinks daily or weekly								
No			14.4	[14.2, 14.5]			7.3	[7.2, 7.4]
Yes			15.9	[15.5, 16.2]			8.9	[8.6, 9.2]
Presence of overweight/obese women in the HH								
No			12.1	[11.9, 12.2]			6.0	[5.8, 6.1]
Yes			20.5	[20.2, 20.8]			11.3	[11.0, 11.5]

**Note**: HH-Household; PSU-Primary Sampling Unit; ^@^ in the sub-sample households where interview of any eligible woman (15–49 years) was completed.

The prevalence of clustering of diabetes was higher among urban households (9.5%) than rural households (6.8%). The caste-based gradient was steeper in case of diabetes where prevalence of clustering for diabetes was almost double (9.4%) among households where household heads belonged to other castes than the households where heads belonged to ST (5.0%). The prevalence of clustering of diabetes varied less by religious groups compared to the prevalence of clustering of hypertension. The prevalence of clustering of diabetes was highest among Christians (10.1%) and lowest among households headed by members who are neither Hindu, Muslim, Sikh or Christian (6.0%). The prevalence of clustering of diabetes increased with increase in the household wealth quintile; the prevalence of clustering of diabetes almost tripled from around 4.2% among households in lowest wealth quintile to 11.6% in highest wealth quintile. The prevalence of clustering of diabetes among households with only one-member consuming alcohol and three or more members consuming alcohol was 6.6% and 11.1%, respectively. Likewise, the prevalence of clustering for diabetes increased from 6.5% among households with only one member using tobacco to 11.5% among households with three or more members using tobacco. Higher prevalence of diabetes clustering was seen in households consuming milk or milk products (8.1%), fish (9.1%), meat or chicken (8.7%), fried food (8.3%), and aerated drinks (8.9%) on a daily or weekly basis.

Higher prevalence of clustering of hypertension (20.5%) and diabetes (11.3%) was seen in households where any woman age 15–49 was overweight or obese.

**Fig 2:**
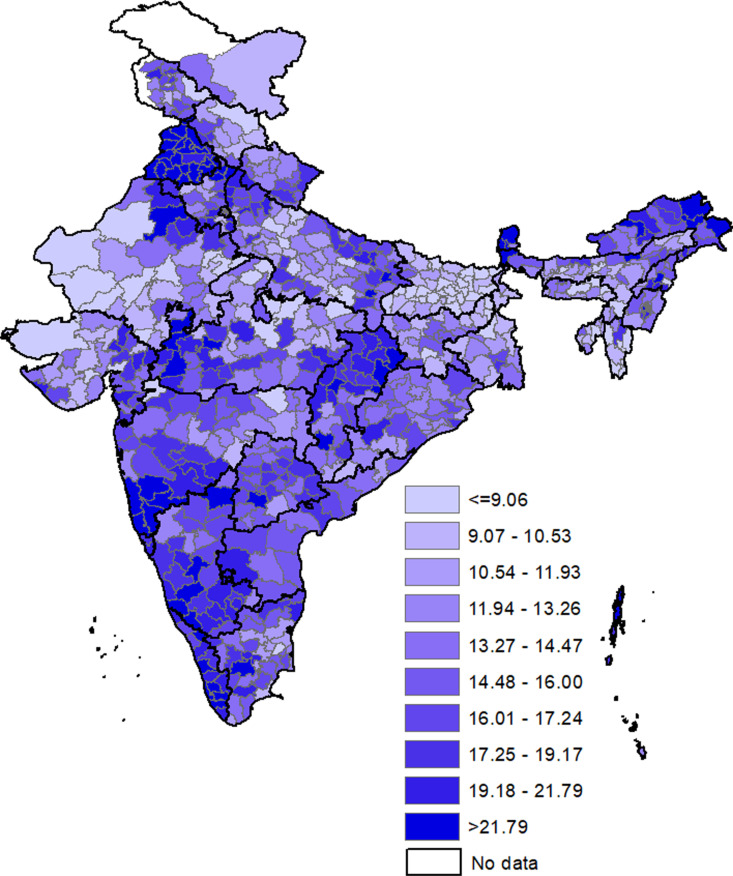
Spatial distribution of households with clustering of hypertension. The base map can be found at https://globalsolaratlas.info/download/india.

**Fig 3:**
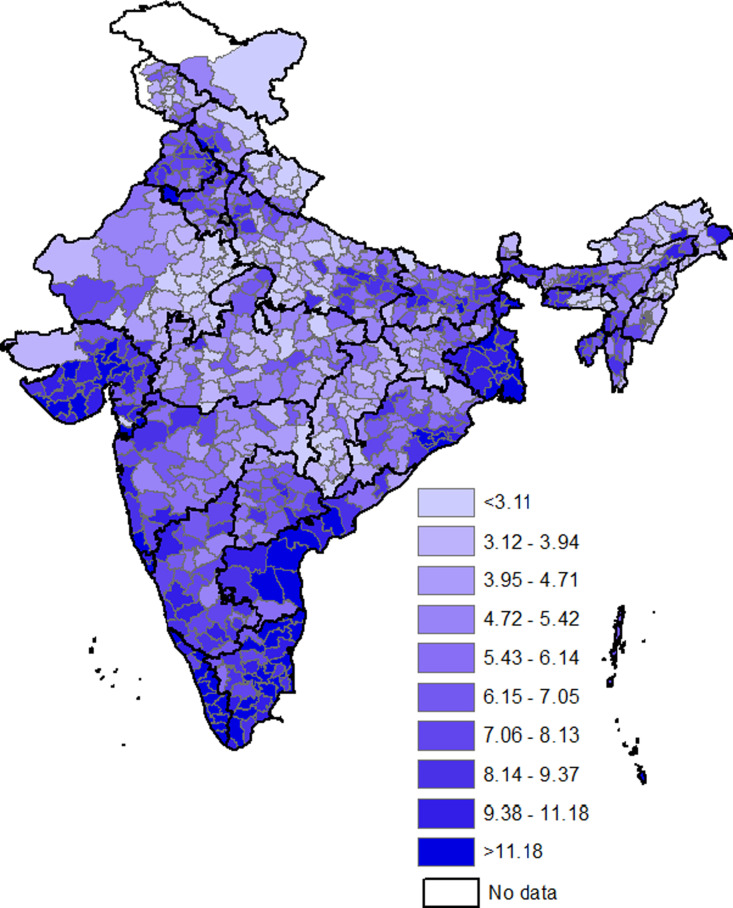
Spatial distribution of households with clustering of diabetes. The base map can be found at https://globalsolaratlas.info/download/india.

Figs 2 and 3 show the spatial distribution of households with clustering of hypertension and diabetes across the districts in India, respectively (the district wise prevalence can be seen in S3 Table). The proportion of households with clustering for hypertension ranged from around 4.0% in Kaushambi (Uttar Pradesh) to 37.3% in Amritsar (Punjab). In about a-third of households in Hoshiarpur, Shahid Bhagat Singh Nagar, Bathinda (All Punjab), West Delhi district (NCT Delhi), Kurukshetra (Haryana), Mahe (Puducherry), and Gurdaspur (Punjab) clustering for hypertension was identified. In particular, many districts in Punjab, Haryana, Kerala, Southern Karnataka, Western Maharashtra, Chhattisgarh, Jharkhand, Sikkim, and Arunachal Pradesh showed higher prevalence (>20%) of clustering of hypertension within households. The clustering of diabetes ranged from about one percent in Kra Daadi (Arunachal Pradesh) to 24.0% in Mahe (Puducherry). Clustering for diabetes was observed in about one-sixth of the households in Pathanamthitta, Kollam, Kottayam, Thrissur, Alappuzha (All Kerala), Morbi (Gujarat), Palakkad, Ernakulam (All Kerala), and Ahmedabad (Gujarat). The districts with higher prevalence (>10%) of clustering within households were observed in almost entire Kerala and Tamil Nadu, Saurashtra, Coastal Karnataka, Andhra Pradesh, Coastal regions of Odisha, and West Bengal.

The district-wise spatial distribution of percentage of households with any member consuming alcohol, using tobacco, daily or at-least weekly consumption of chicken or meat, fried food, fish, curd or milk, aerated drinks, and percentage of households with at-least one woman obese or overweight are shown in S1 Fig. The prevalence of alcohol consumption by any member of the household is high in almost all districts of Telangana, Arunachal Pradesh, Sikkim, Himachal Pradesh, Manipur, and Tripura, many districts in Meghalaya and Uttarakhand, southern and northern districts of Chhattisgarh, southern Jharkhand, western and north-eastern districts of Odisha, eastern Maharashtra, eastern Madhya Pradesh, eastern and hilly districts of Assam, and few districts in Punjab and Jammu & Kashmir. Tobacco use in any form is higher in most of the districts of north-eastern states, Odisha, Chhattisgarh, central districts of Madhya Pradesh, Uttar Pradesh, southern districts of Jharkhand, northern and western districts of West Bengal, peninsular Gujarat, eastern Rajasthan, and eastern region of Maharashtra.

Daily or weekly consumption of chicken or meat by women was higher in most of the States in southern and north-eastern parts of India. Higher meat consumption was also noted in Jammu & Kashmir, Ladakh, western and coastal Maharashtra, eastern Odisha and southern Jharkhand. Consumption of fried food items was particularly seen in eastern and north-eastern parts of India, notably in West Bengal, Odisha, and all north-eastern states. Fried food consumption is also higher in central districts of Uttar Pradesh, Madhya Pradesh, western Rajasthan, western Gujarat, southern Karnataka, central Andhra Pradesh, and many districts of Kerala. The pattern of consumption of fish is somewhat similar to that of chicken or meat except high prevalence of fish consumption along coastal lines of India and in almost all the districts of Odisha, West Bengal, Kerala, and Tamil Nadu. Consumption of milk and milk products was higher in districts in north and western parts of India, Karnataka, Andhra Pradesh, and southern Telangana. Aerated drinks were commonly consumed in north-eastern states of India, Andhra Pradesh, Gujarat, Himachal Pradesh, Karnataka, Jammu & Kashmir, Ladakh, northern districts in Maharashtra, and districts along border of Uttar Pradesh and Bihar. The percentage of households with any woman age 15–49 being overweight/obese is typically higher in northern and southern states of India along with Sikkim, Arunachal Pradesh, and western districts of Maharashtra and Gujarat.

The spatial pattern of higher prevalence of clustering of hypertension within households across districts in India showed good overlap with districts with high use of tobacco and overweight/obesity. The overlap with tobacco use is particularly observed in Chhattisgarh, Odisha, Jharkhand, Madhya Pradesh, coastal Gujarat, eastern Rajasthan, and north-eastern states of India. The overlap with overweight/obesity is typically seen in districts in northern and southern parts of India. The districts with higher prevalence of clustering for diabetes demonstrated good overlap with obesity, consumption of fish, and fried food. The districts with high prevalence of any woman being overweight or obese within household matched very closely with districts with higher prevalence of clustering for diabetes. There is remarkable overlap with fish consumption, seen in almost all districts in southern states, West Bengal, north-eastern states, and Odisha. Overlap with consumption of fried food is seen in central districts of Tripura, Mizoram, West Bengal, Rajasthan, Gujarat, pockets in Madhya Pradesh, and districts along Uttar Pradesh-Bihar border. Overlap of higher prevalence of diabetes clustering was seen with high prevalence of consumption of alcohol in southern, eastern, and north-eastern states whereas to some extent overlap was seen with aerated drinks and diabetes clustering in districts in peninsular Gujarat, central Rajasthan, and districts along Uttar Pradesh-Bihar border.

Household level clustering of hypertension and diabetes was further examined by four-level random intercept logistic regressions; results with complete sample of all households with BP and RBG measurement and subsample of households with BP and RBG measurement and woman’s interview are shown in Tables 4 and 5, respectively. With complete sample of all households with BP and RBG measurements, the estimates of clustering of these diseases within households in null model indicate considerable variations at community, district and state level with intraclass correlation coefficient (ICC) of 0.087 at community, 0.023 at district, and 0.031 at state level for hypertension clustering and ICC of 0.081 at community, 0.026 at district, and 0.052 at state level for diabetes clustering. Community, district and state explained considerable variation in hypertension and diabetes clustering even after inclusion of socio-economic, demographic and residence-related characteristics in the regressions.

**Table 4:** Estimates from the four-level random intercept model.

	2 or more hypertensive members in the HH						2 or more diabetic members in the HH					
	Null Model			Full Model			Null Model			Full Model		
	OR	p-value	95% CI	OR	p-value	95% CI	OR	p-value	95% CI	OR	p-value	95% CI
Number of 15–30 years members in the HH				1.202	<0.001	[1.194, 1.210]				1.183	<0.001	[1.173, 1.195]
Number of 31–50 years members in the HH				1.611	<0.001	[1.596, 1.627]				1.489	<0.001	[1.473, 1.507]
Number of 51–60 years members in the HH				2.636	<0.001	[2.602, 2.670]				2.376	<0.001	[2.339, 2.416]
Number of 61–70 years members in the HH				3.188	<0.001	[3.147, 3.235]				2.715	<0.001	[2.668, 2.765]
Number of 71 + years members in the HH				3.542	<0.001	[3.485, 3.603]				2.663	<0.001	[2.607, 2.723]
% Share of 15 + years female members in the HH				0.996	<0.001	[0.996, 0.997]				0.996	<0.001	[0.996, 0.997]
No. of HH member drink alcohol												
No member (Ref.)												
One member				1.055	<0.001	[1.033, 1.077]				0.972	0.028	[0.944, 0.999]
Two members				1.175	<0.001	[1.134, 1.219]				0.904	0.000	[0.857, 0.953]
Three and more members				1.199	<0.001	[1.130, 1.271]				0.911	0.016	[0.837, 0.989]
No. of HH member smoke and/or use tobacco												
No member (Ref.)												
One member				0.976	0.011	[0.957, 0.997]				0.989	0.203	[0.960, 1.018]
Two members				0.964	0.005	[0.938, 0.991]				1.018	0.180	[0.980, 1.055]
Three and more members				0.879	<0.001	[0.847, 0.914]				0.986	0.298	[0.934, 1.037]
HH head’s education												
No education or below primary (Ref.)												
Primary completed				1.093	<0.001	[1.066, 1.120]				1.070	<0.001	[1.033, 1.108]
Secondary completed				1.126	<0.001	[1.100, 1.151]				1.145	<0.001	[1.110, 1.180]
Higher secondary and above				1.087	<0.001	[1.050, 1.128]				1.168	<0.001	[1.117, 1.219]
HH Wealth Quintile												
Lowest (Ref.)												
Second				1.123	<0.001	[1.092, 1.155]				1.187	<0.001	[1.140, 1.234]
Middle				1.291	<0.001	[1.254, 1.330]				1.420	<0.001	[1.358, 1.480]
Higher				1.495	<0.001	[1.450, 1.545]				1.755	<0.001	[1.678, 1.836]
Highest				1.666	<0.001	[1.602, 1.730]				2.066	<0.001	[1.956, 2.178]
Social group of the HH head												
Scheduled Tribe (Ref.)												
Scheduled Caste				0.966	0.020	[0.935, 0.997]				1.037	0.083	[0.985, 1.090]
Other Backward Class				0.965	0.011	[0.934, 0.995]				1.067	0.003	[1.019, 1.121]
Others				1.009	0.318	[0.974, 1.044]				1.122	<0.001	[1.066, 1.176]
Religion of the HH head												
Other religion (Ref.)												
Hindu				1.006	0.473	[0.949, 1.074]				1.094	<0.001	[1.029, 1.190]
Muslim				1.069	0.020	[1.003, 1.154]				1.124	0.001	[1.038, 1.242]
Christian				1.008	0.413	[0.940, 1.080]				1.041	0.194	[0.954, 1.150]
Sikh				1.156	0.003	[1.044, 1.273]				1.224	<0.001	[1.088, 1.395]
Place of residence												
Urban (Ref.)												
Rural				0.928	<0.001	[0.900, 0.955]				0.946	0.002	[0.910, 0.986]
Proportion of pucca HH in a PSU				1.064	0.001	[1.014, 1.120]				1.094	0.002	[1.027, 1.186]
Constant	0.157	<0.001	[0.146, 0.165]	0.018	<0.001	[0.017, 0.020]	0.061	<0.001	[0.056, 0.065]	0.007	<0.001	[0.006, 0.007]
	*Variance*	*ICC*		*Variance*	*ICC*		*Variance*	*ICC*		*Variance*	*ICC*	
State	0.120	0.031		0.132	0.034		0.203	0.052		0.225	0.058	
District	0.088	0.023		0.097	0.025		0.102	0.026		0.089	0.023	
PSU	0.334	0.087		0.384	0.098		0.317	0.081		0.304	0.078	
Residual	3.293			3.293			3.293			3.293		

**Note**: OR-Odds Ratio; CI-Confidence Interval; ICC-Intra Class Correlation; HH-Household; PSU-Primary Sampling Unit; Ref. Reference category.

**Table 5:** Estimates from four-level random intercept model (sub-sample households where interview of any eligible woman (15-49 years) was completed).

	2 or more hypertensive members in the HH						2 or more diabetic members in the HH					
	Null Model			Full Model			Null Model			Full Model		
	OR	p-value	95% CI	OR	p-value	95% CI	OR	p-value	95% CI	OR	p-value	95% CI
Number of 15–30 years members in the HH				1.214	<0.001	[1.205, 1.222]				1.178	<0.001	[1.165, 1.190]
Number of 31–50 years members in the HH				1.584	<0.001	[1.566, 1.602]				1.445	<0.001	[1.423, 1.465]
Number of 51–60 years members in the HH				2.566	<0.001	[2.530, 2.603]				2.341	<0.001	[2.296, 2.388]
Number of 61–70 years members in the HH				3.046	<0.001	[3.001, 3.094]				2.599	<0.001	[2.547, 2.651]
Number of 71 + years members in the HH				3.313	<0.001	[3.247, 3.378]				2.547	<0.001	[2.486, 2.608]
% Share of 15 + years female members in the HH				0.995	<0.001	[0.995, 0.996]				0.995	<0.001	[0.994, 0.996]
No. of HH member drink alcohol												
No member (Ref.)												
One member				1.055	<0.001	[1.032, 1.080]				0.968	<0.001	[0.938, 0.999]
Two members				1.163	<0.001	[1.116, 1.210]				0.911	0.022	[0.861, 0.964]
Three and more members				1.187	<0.001	[1.119, 1.269]				0.896	<0.001	[0.814, 0.982]
No. of HH member smoke and/or use tobacco												
No member (Ref.)												
One member				0.983	<0.001	[0.962, 1.005]				1.003	<0.001	[0.975, 1.033]
Two members				0.958	0.067	[0.930, 0.987]				1.020	0.425	[0.980, 1.068]
Three and more members				0.887	0.002	[0.851, 0.927]				0.999	0.174	[0.950, 1.054]
HH head’s education												
No education or below primary (Ref.)												
Primary completed				1.044	<0.001	[1.015, 1.073]				1.037	<0.001	[1.001, 1.076]
Secondary completed				1.065	0.001	[1.040, 1.094]				1.081	0.024	[1.047, 1.115]
Higher secondary and above				1.022	<0.001	[0.983, 1.064]				1.101	<0.001	[1.044, 1.155]
HH Wealth Quintile												
Lowest (Ref.)												
Second				1.111	<0.001	[1.075, 1.145]				1.132	<0.001	[1.080, 1.185]
Middle				1.226	<0.001	[1.184, 1.270]				1.292	<0.001	[1.230, 1.359]
Higher				1.379	<0.001	[1.327, 1.432]				1.536	<0.001	[1.456, 1.624]
Highest				1.506	<0.001	[1.441, 1.573]				1.744	<0.001	[1.646, 1.854]
Social group of the HH head												
Scheduled Tribe (Ref.)												
Scheduled Caste				0.944	<0.001	[0.907, 0.983]				1.006	<0.001	[0.949, 1.059]
Other Backward Class				0.958	0.002	[0.922, 0.997]				1.041	0.396	[0.983, 1.094]
Others				0.988	0.019	[0.951, 1.033]				1.093	0.082	[1.030, 1.154]
Religion of the HH head												
Other religion (Ref.)												
Hindu				0.979	<0.001	[0.917, 1.051]				1.084	<0.001	[0.995, 1.195]
Muslim				1.000	0.259	[0.919, 1.081]				1.095	0.038	[0.991, 1.222]
Christian				0.974	0.478	[0.905, 1.053]				1.027	0.048	[0.922, 1.149]
Sikh				1.158	0.244	[1.046, 1.291]				1.204	0.338	[1.027, 1.413]
Place of residence												
Urban (Ref.)												
Rural				0.940	<0.001	[0.909, 0.974]				0.951	<0.001	[0.914, 0.987]
Proportion of pucca HH in a PSU				1.037	<0.001	[0.977, 1.104]				1.089	0.005	[1.016, 1.168]
HH consume milk or milk product daily or weekly												
No (Ref.)												
Yes				0.963	<0.001	[0.94, 0.986]				1.070	<0.001	[1.035, 1.106]
HH consume fish daily or weekly												
No (Ref.)												
Yes				1.025	<0.001	[0.997, 1.053]				1.013	<0.001	[0.979, 1.050]
HH consume chicken or meat daily or weekly												
No (Ref.)												
Yes				0.990	<0.001	[0.964, 1.015]				0.995	<0.001	[0.963, 1.028]
HH consume fried foods daily or weekly												
No (Ref.)												
Yes				1.011	<0.001	[0.992, 1.032]				1.017	<0.001	[0.989, 1.046]
HH consume aerated drinks daily or weekly												
No (Ref.)												
Yes				1.001	<0.001	[0.976, 1.027]				1.030	<0.001	[0.998, 1.064]
Presence of overweight/obese women in the HH												
No (Ref.)												
Yes				1.621	<0.001	[1.587, 1.653]				1.558	<0.001	[1.517, 1.604]
Constant	0.150	<0.001	[0.138, 0.160]	0.021	<0.001	[0.020, 0.022]	0.058	<0.001	[0.052, 0.066]	0.008	<0.001	[0.007, 0.009]
	*Variance*	*ICC*		*Variance*	*ICC*		*Variance*	*ICC*		*Variance*	*ICC*	
State	0.125	0.032		0.122	0.031		0.191	0.049		0.217	0.055	
District	0.095	0.025		0.104	0.026		0.105	0.027		0.091	0.023	
PSU	0.350	0.091		0.399	0.102		0.332	0.085		0.328	0.084	
Residual	3.293			3.293			3.293			3.293		

**Note**: OR-Odds Ratio; CI-Confidence Interval; ICC-Intra Class Correlation; HH-Household; PSU-Primary Sampling Unit; Ref.- Reference category.

A number of socio-economic, demographic and residence-related characteristics of the households were associated with clustering of hypertension and diabetes. The odds of clustering of hypertension increased with the increase in number of household members in each category with higher odds observed in older age groups. The age-specific odds were 1.202 (95% CI: 1.194-1.210) for the age group 15–30 years, 1.611 (95% CI: 1.596-1.627) for the age group 31–50 years, 2.636 (95% CI: 2.602-2.670) for the age group 51–60 years, 3.188 (95% CI: 3.147-3.235) for the age group 61–70 years, and 3.542 (95% CI: 3.485-3.603) for the age group 71 years and above. The odds of clustering of hypertension increased with the increase in number of household members consuming alcohol. For example, a household with three or more members consuming alcohol was 1.199 (95% CI: 1.130-1.271) times as likely as a household with no member consuming alcohol to have clustering of hypertension. In case of tobacco use, a household with three or more members using tobacco was less likely than a household with no member using tobacco to have clustering of hypertension, odds were 0.879 (95% CI: 0.847-0.914) times as likely as a household with no member using tobacco to have clustering of hypertension. The odds of hypertension clustering were positively associated with the education of the household head. Households with head having secondary education or higher secondary and above education were respectively 1.126 (95% CI: 1.100-1.151) and 1.087 (95% CI: 1.050-1.128) times as likely as households with head having no or below primary education to have clustering of hypertension. Clustering of hypertension increased consistently with increase in household wealth. Compared to lowest wealth quintile households, the odds of clustering of hypertension was 1.123 (95% CI: 1.092-1.155), 1.291 (95% CI: 1.254-1.330), 1.495 (95% CI: 1.450-1.545) and 1.666 (95% CI: 1.602-1.730) times higher among second, middle, fourth and highest wealth quintile households, respectively. With respect to caste of the household head, the odds of clustering of hypertension were slightly lower among households where heads belonged to SC and OBC (odds ratio-0.966, 95% CI: 0.935-0.997 for SC and odds ratio 0.965, 95% CI: 0.934-0.995 for OBC) as compared those belonging to ST. The odds of clustering within households were lower in rural (odds ratio – 0.928; 95% CI: 0.900-0.955) than in urban areas. Increase in pucca houses in the community increased the odds (odds ratio – 1.064; 95% CI: 1.014-1.120) of clustering of hypertension.

Similar associations were observed for clustering of diabetes. The clustering of diabetes also increased with increase in the increase in number of household members in each category with higher odds observed in older age groups. The age-specific odds were 1.183 (95% CI: 1.173-1.195) for the age group 15–30 years, 1.489 (95% CI: 1.473-1.507) for the age group 31–50 years, 2.376 (95% CI: 2.339-2.416) for the age group 51–60 years, 2.715 (95% CI: 2.668-2.765) for the age group 61–70 years, and 2.663 (95% CI: 2.607-2.723) for the age group 71 years and above. Interestingly, the odds of clustering of diabetes decreased with increase in number of household members consuming alcohol; a household with three or more members consuming alcohol was 0.911 (95% CI: 0.837-0.989) times as likely as a household with no member consuming alcohol to have clustering of diabetes. Clustering for diabetes within households did not vary much by number of members using tobacco in any form. Like hypertension clustering, the odds of diabetes clustering were positively associated with the education of the household head. As compared to households with heads having no education or less than primary education, the households with heads having primary education were 1.070 (95% CI: 1.033-1.108) times, those with secondary education were 1.145 (95% CI: 1.110-1.180) times, and those with higher secondary or higher education were 1.168 (95% CI: 1.117-1.219) times more likely to have diabetes clustering. Clustering of diabetes steadily increased with increase in household wealth. With reference to lowest wealth quintile households, the odds of clustering of diabetes were 1.187 (95% CI: 1.140-1.234), 1.42 (95% CI: 1.358-1.480), 1.755 (95% CI: 1.678-1.836) and 2.066 (95% CI: 1.956-2.178) times higher among second, middle, fourth and highest wealth quintile households, respectively. The odds for diabetes clustering varied by caste of the head of the household. Households headed by OBC and other castes were more likely than households headed by ST to have clustering of hypertension (odds ratio-1.067, 95% CI: 1.019-1.121 and odds ratio-1.122, 95% CI: 1.066-1.176, respectively). Households with heads belonging to the Sikh religion were more likely to experience clustering of both hypertension and diabetes compared to households headed by individuals of other religions (odds ratio: 1.156, 95% CI: 1.044-1.273 for hypertension, and odds ratio: 1.224, 95% CI: 1.088-1.395 for diabetes). The odds of clustering of diabetes were lower among households residing in rural areas (odds ratio – 0.944; 95% CI: 0.912-0.976) than in urban areas. The likelihood of diabetes clustering in households increased with increase in number of pucca households in the community (odds ratio – 1.094; 95% CI: 1.027-1.186).

With second set of sample of households with BP and RBG measurement and woman’s interview, the variations at community, district, and state level for estimates of clustering of hypertension and diabetes within households slightly changed with ICC of 0.091 at community, 0.025 at district, and 0.032 at state level in the null model for hypertension clustering and ICC of 0.085 at community, 0.027 at district, and 0.049 at state level in the null model for diabetes clustering.

Daily or weekly consumption of fish (odds ratio - 1.025, 95% CI: 0.997-1.104 for hypertension clustering and odds ratio - 1.013, 95% CI: 0.979-1.050 for diabetes clustering) and fried food (odds ratio – odds ratio - 1.011, 95% CI: 0.992-1.032 for hypertension clustering and odds ratio - 1.017, 95% CI: 0.989-1.046 for diabetes clustering) were positively associated with clustering of these two NCDs at the household level. Daily or weekly consumption of milk or milk products (odds ratio - 1.070, 95% CI: 1.035-1.106) and aerated drinks (odds ratio - 1.030, 95% CI: 0.998-1.046) was positively associated with diabetes clustering only. The odds of clustering of hypertension as well as diabetes increased with the presence of any overweight or obese woman age 15–59 in the household (odds ratio - 1.621, 95% CI: 1.587-1.653 for hypertension clustering and odds ratio - 1.558, 95% CI: 1.517-1.604 for diabetes clustering). After inclusion of these variables, odds of hypertension and diabetes clustering by education, wealth status, caste and religion were slightly reduced, however the direction of association remained unchanged.

The ICCs from the full model indicate that substantial proportion of variations in the prevalence of clustering of hypertension and diabetes within households was due to the community (10.2% for hypertension and 8.4% for diabetes), district (2.6% for hypertension and 2.3% for diabetes), and state (3.1% for hypertension and 5.5% for diabetes).

## Discussion

Our study is perhaps the first to examine the distribution of hypertension and diabetes in the context of clustering of these diseases within households at the district-level using a large-scale nationally representative household survey. Both hypertension and diabetes exhibited considerable clustering. Two or more members of a household were identified with hypertension in 14.9% of households and with diabetes in 7.7% households in India. Further hypertension and diabetes were disproportionately concentrated within these clustered households. The 14.9% households with hypertension clustering harboured about half of the total cases of hypertension in India and 7.7% households with diabetes clustering harboured about 40% of total cases of diabetes in India. Though quantitative estimates per se have not been previously reported, available literature provides evidence of unequal distribution of NCDs and clustering of risk factors within households [1,3,25,26,56,57]. Given that the large amount of disease burden is nested within the clustered households, proper understanding of the epidemiology of hypertension and diabetes in India and efficient planning of health care for these diseases warrants providing due diligent attention to the phenomenon of clustering.

Huge variation was seen in the prevalence of clustering across the 707 districts of India. The prevalence of hypertension clustering varied from around 3.9% in Kaushambi to around 37.3% in Amritsar and that for diabetes clustering varied from 1.4% in Kra Daadi to 25.0% in Mahe. The spatial distribution of clustering of hypertension and diabetes also demonstrated distinct pockets of high prevalence of clustering. Prioritizing and targeting such areas with high case burden through intensified surveillance, health education campaigns, and community-based screening programmes and efforts for improved disease management can yield better results and accelerate progress towards Sustainable Development Goal 3.4 (SDG 3.4). Meanwhile, all regions can be targeted with preventive interventions, such as health education drives, to raise awareness about risk factors and promote lifestyle modifications for healthier behaviours.

Remarkably, the spatial pattern of districts with higher clustering of hypertension and diabetes overlapped quite well with spatial patterns of districts having high prevalence of various risk factors like alcohol consumption, tobacco use, overweight or obesity and consumption of various types of food items providing potential explanations for clustering in different regions.

The higher clustering of diabetes and hypertension in coastal areas could be due to the greater consumption of fish and fried food. Similarly, the regions with higher clustering of hypertension and diabetes in southern, northern, and western regions can be due to higher prevalence of overweight or obesity. Higher clustering for hypertension in central and north-eastern parts of India can be due to higher prevalence of use of tobacco whereas higher clustering in Goa and northern states like Punjab and Himachal Pradesh can be due higher consumption of alcohol. Higher clustering of diabetes in southern, eastern, and north-eastern states can be due to higher alcohol consumption whereas that in Saurashtra region of Gujarat and westerns districts of Bihar can be due to higher consumption of aerated drinks.

The observations from spatial patterns were very well supported by findings from the random intercept models. The regressions at national level revealed that the odds for clustering of hypertension increased with increase in number of household members engaged in consumption of alcohol. Clustering of both the diseases was positively associated with presence of any overweight or obese woman in the household, which can serve as a proxy for other household members’ BMI, given the documented concordance of high BMI within couples [16,39,58,59]. The corroboration of diabetes clustering along India’s coastline and adjoining states and higher consumption of fish and fried food in the same region is particularly notable. This is in accordance with existing literature associating fried fish consumption with development of type II diabetes [60–63]. Fish consumption was also associated with hypertension clustering, consistent with evidence supporting association of hypertension with consumption of dried fish, also a common practice in India [61,64,65]. However, the NFHS-5 questionnaire did not collect separate information on consumption of dried fish, making it difficult to draw any such correlation. These associations, though interesting, demand further investigation. These findings can strengthen public health interventions and policies by providing key insights about specific risk factors driving clustering in different regions to effectively formulate region-specific interventions.

Higher BMI is one of the key factor contributing to the clustering of hypertension and clustering of diabetes, highlighting the urgent need for nationwide efforts to encourage healthy dietary habits, regular physical activity, and overall lifestyle improvements. In addition, interventions to reduce alcohol and tobacco use are essential. Our findings call for region-specific interventions, particularly in coastal regions, southern, and eastern states, where focus can be on reducing consumption of fried food and fried fish, while promoting healthier recipes of fish. The association between fish consumption and clustering of hypertension is likely due to consumption of dried fish, which is high in salt. This can be taken up for targeted interventions, particularly along India’s coastline. In states such as Gujarat and Andhra Pradesh, efforts should focus on reducing the intake of sugary aerated drinks and promoting traditional, healthier beverage options like buttermilk.

The clustering for hypertension as well as diabetes within household was positively and significantly associated with number of members in the household and particularly the number of older members, household wealth quintile, urban residence, households where heads belonged to Sikh religion and OBC or other castes, and higher education of the head of the households. Overall similar associations were observed for disease prevalence among individuals as well as concordant couples [21–23,38,43]. Population ageing is on the rise in India, particularly pronounced in the southern states where higher prevalence of clustering is noticed [66]. With ageing population, the clustering phenomenon is expected to intensify. Such clustering is likely to put tremendous strain on these households, increasing demands for healthcare, social support, and financial resources to cover all care-related costs.

Though overall individual prevalence of hypertension and diabetes is lower in rural areas than urban areas and the likelihood of clustering of hypertension and diabetes within households is lower in rural areas, given the large share of India’s population dwelling in rural areas, evidence of clustering of hypertension and diabetes in rural households poses a big challenge for health systems to deliver health care in rural areas as advanced interventions, diagnostics, and expertise will be potentially required to manage these conditions, prevent complications, and ultimately to prevent mortality. The health care system needs to be adequately bolstered to deliver services for NCDs in rural areas. Our finding also underscores the need to integrate specialised care with primary health care and expansion of outreach of specialised care by various means. Low level of education among elderly population in India further exacerbates the vulnerability of the clustered households to complications of these diseases [48]. This is due to lack of awareness about diseases, inability to identify complications, and difficulties in seeking, accessing, and adhering to treatment. To address this issue, initiative for creating awareness and imparting relevant knowledge must be tailored to suit to the specific needs of the target audience and should be effectively implemented.

The odds for clustering of hypertension and diabetes was more among households belonging to higher wealth quintiles. While wealthier households are more likely to be aware about hypertension and diabetes and seek treatment, even among this group the awareness, treatment and control of these conditions is far from satisfactory [8,9]. This suggests there is considerable potential for improved health outcomes through targeted awareness, treatment and control activities even among wealthier households. The situation is potentially more drastic for households belonging to deprived sections of the society. Although these households exhibit lower clustering prevalence, when affected, they are likely to have lower awareness, treatment and control [8,9,67]. Failure to promptly manage hypertension and diabetes may eventually lead to disproportionate clustering of deaths due to cardiovascular events and other complications within clustered households.

The interactions and interplay of various factors contributing to clustering and the variations in amount of clustering at community, district, and state level was analysed using random intercept regressions. The results demonstrate considerable inequality; hypertension and diabetes clustering being affected by community and the state. The ICC for hypertension and diabetes was highest at the community level indicating the highest impact of factors in the immediate neighbourhood like common shared environment, similar food patterns, settlement patterns, and socio-cultural practices associated with high risk behaviours. These findings are in accordance with previous studies that have suggested influence of immediate neighbourhood on risks associated with NCDs, though evidence from India is limited [68–70].

The existence of clustering of hypertension and diabetes within households and high impact of factors in the immediate vicinity of households, supports the relevance of the role of social networks in disease transmission. Therefore, gaining a better understanding of the dynamics of social transmission of diseases across the social ties may aid in utilizing the same social ties for better diagnosis, treatment, and prevention of complications of hypertension and diabetes [28,30–32,71,72]. Though we did not explore this phenomenon in our study, exploring disease transmission across social ties within and beyond family networks in the unique socio-cultural context of India is certainly a topic of interest.

The present study has some limitations. We identified diabetes based on values of random blood glucose which are less reliable than glycated haemoglobin or fasting blood glucose. Collecting these biomarkers in NFHS like survey with vast geographical expanse and large sample size have operational and economic limitations. Random blood glucose is a sensitive and specific biomarker for screening of diabetes and the cut-off used in our study >140 mg/dL corresponds with the HbA1c 6.5% [73,74]. Though technically it was feasible to segregate respondents with 8 hours fasting, we did not attempt it because NFHS-5 was not designed for fasting blood collection like other DHS surveys where respondents were prior informed to remain fasted and later blood glucose was tested [75]. Also, segregating fasting individuals would reduce sample size, particularly affecting the within household analysis. The reported consumption of various food items can be affected by recall bias. Food consumption in our analysis is based on the self-reported dietary data which can be affected by social desirability or social approval. However, this is less likely to happen in NFHS as the questionnaires in NFHS are developed based on years of experience of DHS surveys across countries. The questions are standardized and the interviews are conducted in privacy by thoroughly trained interviewers. These data have been widely utilized for dietary analysis over the various rounds of NFHS. BMI information was not available for all adult members of the household and we considered woman’s BMI as proxy for all other members. NFHS being a cross sectional survey, the scope for any causal analysis is limited. The age of onset of hypertension and diabetes are not available in NFHS-5 limiting any additional analysis. Although physical activity, dietary diversity, and salt consumption are important determinants of hypertension and diabetes clustering, we were unable to include them in our analysis due to the lack of these data in NFHS-5.

The analysis based on complete sample and sub-sample may not be strictly comparable due to difference in the age-structure of the members. However, this is less problematic given that the mean age of members in the full sample (39.5 years) was only 2 years more than that of the sub-sample (37.5 years). While we found association of fish consumption with hypertension, we could not differentiate whether it was consumption of dried fish as this information was not collected in NFHS-5.

Our study findings provide crucial insights about district-wise distribution of hypertension and diabetes in India with a unique context of clustering of these diseases within households using large-scale nationally representative survey. We emphasize high priority areas for intensified interventions aimed at raising awareness, rapid case detection, and adequate management of hypertension and diabetes to ensure rapid advancements towards SDG 3.4. Taking cognizance of clustering of NCDs is particularly important in low resource settings, such as LMICs, where identifying and focusing on high priority areas can yield higher dividends. Our study quantitatively estimates the clustering within households, providing empirical evidence in support of family level interventions for efficient and rapid management of hypertension and diabetes. The varied case burden across different districts and regions, as indicated by prevalence of clustered households, strongly advocates for customised district- and region-specific policy formulation. The distinct spatial distribution patterns of clustering of hypertension and diabetes aligned with the distribution of overweight or obesity, consumption of alcohol and tobacco, and consumption of fish and fried food, suggesting important region-specific associations with clustering, which can be further evaluated and utilized to plan interventions for prevention, education, screening, and treatment. The evidence generated by our study should also be useful in exploring the potential of community-based interventions for management of hypertension and diabetes in India. Additionally, our study offers recommendations for The DHS programme. DHS being a valuable dataset on NCDs and their risk factors for evidence-based policy making for many developing countries, should collect information on weight and height measurement for all household members aged 15 years and above, in addition to blood pressure and blood glucose measurement. DHS should also strive to collect relevant information on NCDs, such as daily salt consumption, the timing of onset or diagnosis of hypertension and diabetes, and levels of physical activity. Further, collection of community data in DHS surveys may enrich analysis of the NCDs. Our analytical approach can serve as a model for other LMICs, where DHS or similar surveys are conducted at regular intervals. By adapting to our analysis, these countries may gain deeper insights into the epidemiology of hypertension and diabetes within their specific contexts.

## Supporting information

S1 Checklist STROBE checklist of items that should be included in reports of cross-sectional studies. (DOCX)

S1 Text Measurement of weight and height and calculation of BMI. (DOCX)

S2 Text Outcome definitions. (DOCX)

S1 Fig District-wise spatial distribution of percentage of households with any member consuming alcohol, using tobacco, daily or at-least weekly consumption of chicken or meat, fried food, fish, curd or milk, aerated drinks, and percentage of households with at-least one woman obese or overweight. (TIF)

S1 Table Missing observations on blood pressure measurement and selected household characteristics. (DOCX)

S2 Table Missing observations on blood sugar measurement and selected household characteristics. (DOCX)

S3 Table District-wise prevalence of clustering of hypertension and clustering of diabetes within the households (HH) in India. (DOCX)
