# Supplementary material for: Clustering of hypertension and clustering of diabetes within households across districts of India: A cross-sectional analysis using a nationally representative household survey
Source: PLOS Glob Public Health. 2025 Jun 17;5(6):e0004648. doi: 10.1371/journal.pgph.0004648 (PMC12173236; doi:10.1371/journal.pgph.0004648)
Supplement: S1 Checklist — (DOCX) [file pgph.0004648.s001.docx]

**S1 STROBE Checklist.** STROBE checklist of items that should be included in reports of cross-sectional studies

|  | Item No | Recommendation | Section, paragraph Response |
| --- | --- | --- | --- |
| **Title and abstract** | 1 | (*a*) Indicate the study’s design with a commonly used term in the title or the abstract | Title |
|  |  | (*b*) Provide in the abstract an informative and balanced summary of what was done and what was found | Abstract: Methods & findings. Author summary: Why was this study was done? What did the researchers do and find? |
| Introduction | | |  |
| Background/ rationale | 2 | Explain the scientific background and rationale for the investigation being reported | Abstract: Background. Author summary: Why was this study done? Introduction |
| Objectives | 3 | State specific objectives, including any prespecified hypotheses | Abstract: Background. Introduction: final paragraph. |
| Methods | | |  |
| Study design | 4 | Present key elements of study design early in the paper | Abstract: Methods and findings. Methods: Data Source, Measurement, Analytical procedure |
| Setting | 5 | Describe the setting, locations, and relevant dates, including periods of recruitment, exposure, follow-up, and data collection | Methods: Data Source, Measurement, S1 Text |
| Participants | 6 | (*a*) Give the eligibility criteria, and the sources and methods of selection of participants | Methods: Data Source, Measurement, Analytical procedure. |
| Variables | 7 | Clearly define all outcomes, exposures, predictors, potential confounders, and effect modifiers. Give diagnostic criteria, if applicable | Methods: Data Source, Measurement, Analytical procedure. S2 Text |
| Data sources/ measurement | 8* | For each variable of interest, give sources of data and details of methods of assessment (measurement). Describe comparability of assessment methods if there is more than one group | Methods: Data Source, Measurement, Analytical procedure. S1 Text, S2 Text |
| Bias | 9 | Describe any efforts to address potential sources of bias | Methods: Data Source, Measurement, Analytical procedure. |
| Study size | 10 | Explain how the study size was arrived at | Methods: Data Source, Measurement, Analytical procedure. Results: first paragraph, Fig 1 |
| Quantitative variables | 11 | Explain how quantitative variables were handled in the analyses. If applicable, describe which groupings were chosen and why | Methods: Analytical procedure, Table 1 |
| Statistical methods | 12 | (*a*) Describe all statistical methods, including those used to control for confounding | Methods: Analytical procedure |
|  |  | (*b*) Describe any methods used to examine subgroups and interactions | Methods: Analytical procedure |
|  |  | (*c*) Explain how missing data were addressed | Methods: Data Source second paragraph |
|  |  | (*d*) If applicable, describe analytical methods taking account of sampling strategy | Methods: Analytical procedure last paragraph |
|  |  | (*e*) Describe any sensitivity analyses | Not applicable |
| Results | | |  |
| Participants | 13 | (a) Report numbers of individuals at each stage of study—eg numbers potentially eligible, examined for eligibility, confirmed eligible, included in the study, completing follow-up, and analysed | Fig 1, Results: First paragraph |
|  |  | (b) Give reasons for non-participation at each stage | Fig 1, Results: First paragraph |
|  |  | (c) Consider use of a flow diagram | Fig 1 |
| Descriptive data | 14* | (a) Give characteristics of study participants (eg demographic, clinical, social) and information on exposures and potential confounders | Table 1 |
|  |  | (b) Indicate number of participants with missing data for each variable of interest | S1 Table and S2 Table |
| Outcome data | 15* | Report numbers of outcome events or summary measures | Table 1, Table 2, and Table 3 |
| Main results | 16 | (*a*) Give unadjusted estimates and, if applicable, confounder-adjusted estimates and their precision (eg, 95% confidence interval). Make clear which confounders were adjusted for and why they were included | Table 2, Table 3 |
|  |  | (*b*) Report category boundaries when continuous variables were categorized | Tables 1 |
|  |  | (*c*) If relevant, consider translating estimates of relative risk into absolute risk for a meaningful time period | Not Applicable |
| Other analyses | 17 | Report other analyses done—eg analyses of subgroups and interactions, and sensitivity analyses | Not Applicable |
| Discussion | | |  |
| Key results | 18 | Summarise key results with reference to study objectives | Abstract: Methods and findings, Conclusions. Discussion: paragraphs 1-9, 11 |
| Limitations | 19 | Discuss limitations of the study, taking into account sources of potential bias or imprecision. Discuss both direction and magnitude of any potential bias | Abstract: Methods and findings, Discussion: paragraph 10 |
| Interpretation | 20 | Give a cautious overall interpretation of results considering objectives, limitations, multiplicity of analyses, results from similar studies, and other relevant evidence | Discussion |
| Generalis-ability | 21 | Discuss the generalisability (external validity) of the study results | Discussion: final paragraph (11) |
| Other information | | |  |
| Funding | 22 | Give the source of funding and the role of the funders for the present study and, if applicable, for the original study on which the present article is based | Provided during submission process |
