# Supplementary material for: Clustering of hypertension and clustering of diabetes within households across districts of India: A cross-sectional analysis using a nationally representative household survey
Source: PLOS Glob Public Health. 2025 Jun 17;5(6):e0004648. doi: 10.1371/journal.pgph.0004648 (PMC12173236; doi:10.1371/journal.pgph.0004648)
Supplement: S1 Text — (DOCX) [file pgph.0004648.s002.docx]

**S1 Text**: Measurement of weight and height and calculation of BMI

In NFHS-5, weight and height of women age 15-49 years were measured after completion of their individual interview. Weight was measured using Seca 874 weighing scale and height was measured using Seca 217 stadiometer manufactured by Seca GmbH & Co. KG, Germany. Body mass index (BMI) was calculated as weight of a person measured in kilograms divided by squared height of that person measured in meters. Respondents with body mass index (BMI) >/=25 kg/m^2^ were considered as Overweight/Obese.

The detailed procedure for weight and height measurement in NFHS-5 can be found in the biomarker manual of NFHS-5 available at <https://www.nfhsiips.in/nfhsuser/manual.php>
