# Supplementary material for: Clustering of hypertension and clustering of diabetes within households across districts of India: A cross-sectional analysis using a nationally representative household survey
Source: PLOS Glob Public Health. 2025 Jun 17;5(6):e0004648. doi: 10.1371/journal.pgph.0004648 (PMC12173236; doi:10.1371/journal.pgph.0004648)
Supplement: S2 Text — (DOCX) [file pgph.0004648.s003.docx]

**S2 Text**: Outcome definitions

| **Outcome** |  | **Definition** |
| --- | --- | --- |
| Hypertension | 1 | 1. SBP ≥140 mm Hg OR DBP ≥ 90 mm Hg, or 2. reported currently taking prescribed medication to control BP |
|  | 0 | Otherwise |
| Diabetes | 1 | 1. RBG >140 mg/dL, or 2. reported currently taking prescribed medication to control blood glucose |
|  | 0 | Otherwise |
| Clustering | 1 | Household-level clustering for a disease occurs when a household has two or more members identified with that particular disease |
|  | 0 | When a household has less than two members identified with a particular disease |
| Body Mass Index (BMI) | Weight (kg)/ Height (m)^2^ | |
| Overweight/obese | BMI ≥ 25 kg/m^2^ | |
| **Note**: SBP = systolic blood pressure, DBP = diastolic blood pressure, RBG = Random Blood Glucose | | |
