# Supplementary material for: Clustering of hypertension and clustering of diabetes within households across districts of India: A cross-sectional analysis using a nationally representative household survey
Source: PLOS Glob Public Health. 2025 Jun 17;5(6):e0004648. doi: 10.1371/journal.pgph.0004648 (PMC12173236; doi:10.1371/journal.pgph.0004648)
Supplement: S2 Table — (DOCX) [file pgph.0004648.s006.docx]

**S2 Table**: Missing observations on blood sugar measurement and selected household characteristics

|  | **n = 636,691** | |  | **Analytical**  **Sample**  **(n = 615,125)** | |  | **n = 636,691** | |  | **Analytical**  **Sample**  **(n = 497,949)** | |
| --- | --- | --- | --- | --- | --- | --- | --- | --- | --- | --- | --- |
|  | **Missing**  **cases** | **%** |  | **Missing**  **cases** | **%** |  | **Missing**  **cases** ^@^ | **%** |  | **Missing**  **cases** ^@^ | **%** |
| Blood sugar * | 21,566 | 3.51 |  | 0 | 0 |  | 21,566 | 3.51 |  | 0 | 0 |
| Age | 0 | 0 |  | 0 | 0 |  | 0 | 0 |  | 0 | 0 |
| No. of HH members drink alcohol | 0 | 0 |  | 0 | 0 |  | 0 | 0 |  | 0 | 0 |
| No. of HH members smoke and/or use tobacco | 0 | 0 |  | 0 | 0 |  | 0 | 0 |  | 0 | 0 |
| HH head's education | 0 | 0 |  | 0 | 0 |  | 0 | 0 |  | 0 | 0 |
| HH Wealth Quintile | 0 | 0 |  | 0 | 0 |  | 0 | 0 |  | 0 | 0 |
| Social group of the HH head | 0 | 0 |  | 0 | 0 |  | 0 | 0 |  | 0 | 0 |
| Religion of the HH head | 0 | 0 |  | 0 | 0 |  | 0 | 0 |  | 0 | 0 |
| Place of residence | 0 | 0 |  | 0 | 0 |  | 0 | 0 |  | 0 | 0 |
| Pucca HH | 0 | 0 |  | 0 | 0 |  | 0 | 0 |  | 0 | 0 |
| HH consume fish daily or weekly ^#^ |  |  |  |  |  |  | 126,772 | 19.91 |  | 0 | 0 |
| HH consume chicken or meat daily or weekly ^#^ |  |  |  |  |  |  | 126,772 | 19.91 |  | 0 | 0 |
| HH consume fried foods daily or weekly ^#^ |  |  |  |  |  |  | 126,772 | 19.91 |  | 0 | 0 |
| HH consume aerated drinks daily or weekly ^#^ |  |  |  |  |  |  | 126,772 | 19.91 |  | 0 | 0 |
| Presence of overweight/obese women in the HH ^#^ |  |  |  |  |  |  | 126,772 | 19.91 |  | 0 | 0 |
| **Note**: HH-household; ^@^ in the sub-sample households where interview of any eligible woman (15-49 years) was completed;  * HH where the measurements were not taken is treated as missing;  ^#^ Questions on consumption were asked only to the eligible women (15-49) interviewed in the survey | | | | | | | | | | | |
