# Supplementary material for: Clustering of hypertension and clustering of diabetes within households across districts of India: A cross-sectional analysis using a nationally representative household survey
Source: PLOS Glob Public Health. 2025 Jun 17;5(6):e0004648. doi: 10.1371/journal.pgph.0004648 (PMC12173236; doi:10.1371/journal.pgph.0004648)
Supplement: S3 Table — (DOCX) [file pgph.0004648.s007.docx]

**S3 Table**: District-wise prevalence of clustering of hypertension and clustering of diabetes within the households (HH) in India

| **District** | **2 or more**  **hypertensive**  **members in the**  **HH** | **2 or more**  **diabetic**  **members in the**  **HH** |
| --- | --- | --- |
| Adilabad | 14.3 | 4.6 |
| Agar Malwa | 9.8 | 8.4 |
| Agra | 12.1 | 2.4 |
| Ahmadabad | 12.1 | 14.7 |
| Ahmadnagar | 19.2 | 6.1 |
| Aizawl | 18.1 | 7.2 |
| Ajmer | 8.8 | 2.9 |
| Akola | 15.0 | 5.0 |
| Alappuzha | 25.5 | 16.1 |
| Aligarh | 11.5 | 5.7 |
| Alirajpur | 18.6 | 3.8 |
| Allahabad | 8.3 | 8.3 |
| Almora | 16.2 | 2.9 |
| Alwar | 17.6 | 1.4 |
| Ambala | 30.1 | 5.9 |
| Ambedkar Nagar | 12.0 | 9.5 |
| Amethi | 11.2 | 5.6 |
| Amravati | 14.3 | 6.8 |
| Amreli | 8.6 | 10.5 |
| Amritsar | 37.3 | 7.7 |
| Anand | 21.5 | 11.2 |
| Anantapur | 14.5 | 6.1 |
| Anantnag | 13.4 | 5.7 |
| Anjaw | 24.2 | 9.6 |
| Anugul | 12.0 | 8.1 |
| Anuppur | 16.8 | 6.4 |
| Araria | 6.8 | 8.3 |
| Aravali | 16.1 | 8.8 |
| Ariyalur | 11.6 | 11.3 |
| Arwal | 7.6 | 6.4 |
| Ashoknagar | 10.0 | 5.1 |
| Auraiya | 18.7 | 2.8 |
| Aurangabad | 9.9 | 7.0 |
| Aurangabad | 14.4 | 8.4 |
| Azamgarh | 17.4 | 9.4 |
| Badgam | 17.0 | 4.2 |
| Bagalkot | 16.5 | 7.0 |
| Bageshwar | 15.8 | 2.6 |
| Baghpat | 19.4 | 6.9 |
| Bahraich | 17.3 | 2.9 |
| Baksa | 18.6 | 7.9 |
| Balaghat | 14.7 | 4.5 |
| Balangir | 14.3 | 6.2 |
| Baleshwar | 13.5 | 5.7 |
| Ballia | 16.8 | 8.8 |
| Balod | 20.2 | 3.6 |
| Baloda Bazar | 20.6 | 3.4 |
| Balrampur | 18.0 | 4.0 |
| Balrampur | 18.4 | 3.3 |
| Banas Kantha | 9.2 | 9.1 |
| Banda | 16.6 | 9.0 |
| Bandipore | 15.8 | 5.7 |
| Bangalore | 19.2 | 9.7 |
| Bangalore Rural | 21.5 | 9.1 |
| Banka | 9.7 | 7.4 |
| Bankura | 13.1 | 10.6 |
| Banswara | 11.9 | 4.6 |
| Bara Banki | 13.1 | 4.6 |
| Baramula | 19.5 | 6.8 |
| Baran | 9.4 | 2.4 |
| Bareilly | 9.2 | 4.8 |
| Bargarh | 15.0 | 8.2 |
| Barmer | 6.6 | 7.3 |
| Barnala | 32.6 | 7.6 |
| Barpeta | 11.5 | 9.0 |
| Barwani | 17.9 | 4.7 |
| Bastar | 13.0 | 2.8 |
| Basti | 20.7 | 5.7 |
| Bathinda | 34.2 | 6.2 |
| Baudh | 14.4 | 5.3 |
| Begusarai | 8.0 | 7.4 |
| Belgaum | 16.1 | 7.2 |
| Bellary | 16.0 | 5.0 |
| Bemetara | 23.3 | 3.9 |
| Betul | 12.0 | 5.7 |
| Bhadradri Kothagudem | 19.0 | 8.3 |
| Bhadrak | 13.5 | 8.1 |
| Bhagalpur | 8.0 | 7.9 |
| Bhandara | 13.4 | 4.9 |
| Bharatpur | 15.4 | 3.1 |
| Bharuch | 17.3 | 12.6 |
| Bhavnagar | 12.6 | 9.8 |
| Bhilwara | 9.6 | 3.8 |
| Bhind | 7.9 | 6.3 |
| Bhiwani | 12.8 | 4.6 |
| Bhojpur | 8.1 | 11.0 |
| Bhopal | 10.8 | 6.2 |
| Bid | 21.1 | 5.7 |
| Bidar | 16.7 | 5.1 |
| Bijapur | 10.8 | 3.2 |
| Bijapur | 13.9 | 7.3 |
| Bijnor | 16.9 | 8.0 |
| Bikaner | 8.5 | 5.4 |
| Bilaspur | 12.7 | 11.6 |
| Bilaspur | 18.1 | 4.9 |
| Birbhum | 8.5 | 11.9 |
| Bishnupur | 18.3 | 8.1 |
| Biswanath | 15.4 | 3.3 |
| Bokaro | 16.0 | 5.6 |
| Bongaigaon | 7.4 | 7.9 |
| Botad | 7.8 | 10.6 |
| Budaun | 6.6 | 5.4 |
| Bulandshahr | 14.6 | 8.3 |
| Buldana | 14.5 | 5.1 |
| Bundi | 14.0 | 3.0 |
| Burhanpur | 8.5 | 3.3 |
| Buxar | 8.1 | 6.4 |
| Cachar | 8.6 | 7.8 |
| Central | 21.6 | 6.4 |
| Chamarajanagar | 22.6 | 7.3 |
| Chamba | 11.3 | 4.0 |
| Chamoli | 12.8 | 1.8 |
| Champawat | 15.6 | 4.0 |
| Champhai | 8.6 | 5.2 |
| Chandauli | 13.5 | 5.8 |
| Chandel | 13.8 | 4.2 |
| Chandigarh | 16.3 | 9.7 |
| Chandrapur | 12.5 | 4.3 |
| Changlang | 16.1 | 3.7 |
| Charaideo | 10.8 | 6.8 |
| Charkhi Dadri | 13.5 | 5.5 |
| Chatra | 9.1 | 4.9 |
| Chennai | 19.4 | 13.1 |
| Chhatarpur | 9.6 | 4.7 |
| Chhindwara | 13.5 | 5.2 |
| Chhota Udaipur | 13.4 | 7.2 |
| Chikkaballapura | 17.6 | 5.8 |
| Chikmagalur | 19.2 | 7.4 |
| Chirang | 15.8 | 5.0 |
| Chitradurga | 22.7 | 7.8 |
| Chitrakoot | 10.8 | 3.0 |
| Chittaurgarh | 14.5 | 3.7 |
| Chittoor | 13.3 | 9.7 |
| Churachandpur | 11.8 | 7.7 |
| Churu | 22.7 | 4.1 |
| Coimbatore | 18.6 | 8.5 |
| Cuddalore | 12.3 | 11.6 |
| Cuttack | 15.1 | 9.5 |
| Dadra & Nagar Haveli | 7.0 | 7.1 |
| Dakshin Dinajpur | 9.2 | 11.9 |
| Dakshina Kannada | 23.9 | 7.3 |
| Daman | 8.6 | 6.1 |
| Damoh | 12.4 | 3.1 |
| Dantewada | 13.8 | 4.4 |
| Darbhanga | 10.1 | 6.9 |
| Darjiling | 23.9 | 8.0 |
| Darrang | 7.1 | 7.1 |
| Datia | 10.8 | 6.6 |
| Dausa | 8.9 | 3.8 |
| Davanagere | 17.7 | 9.5 |
| Debagarh | 12.1 | 4.1 |
| Dehradun | 15.8 | 7.1 |
| Deoghar | 9.1 | 5.7 |
| Deoria | 16.7 | 6.4 |
| Devbhumi Dwarka | 13.7 | 9.4 |
| Dewas | 22.1 | 4.0 |
| Dhalai | 6.6 | 6.8 |
| Dhamtari | 16.7 | 6.0 |
| Dhanbad | 13.7 | 7.2 |
| Dhar | 15.7 | 6.7 |
| Dharmapuri | 15.6 | 6.9 |
| Dharwad | 18.9 | 7.3 |
| Dhaulpur | 7.4 | 2.6 |
| Dhemaji | 10.5 | 3.7 |
| Dhenkanal | 13.6 | 5.9 |
| Dhubri | 10.6 | 4.0 |
| Dhule | 16.3 | 8.8 |
| Dibang Valley | 27.2 | 2.9 |
| Dibrugarh | 11.9 | 10.0 |
| Dima Hasao | 10.5 | 4.7 |
| Dimapur | 10.8 | 6.4 |
| Dindigul | 21.4 | 13.8 |
| Dindori | 20.1 | 3.6 |
| Diu | 10.0 | 9.7 |
| Doda | 14.9 | 4.5 |
| Dohad | 13.5 | 6.8 |
| Dumka | 9.2 | 3.8 |
| Dungarpur | 8.9 | 8.6 |
| Durg | 20.6 | 6.6 |
| East | 21.6 | 5.4 |
| East District | 12.8 | 5.2 |
| East Garo Hills | 13.3 | 8.3 |
| East Godavari | 17.1 | 11.8 |
| East Jantia Hills | 9.8 | 2.7 |
| East Kameng | 13.8 | 5.1 |
| East Khasi Hills | 12.9 | 2.5 |
| East Siang | 26.2 | 5.2 |
| Ernakulam | 21.1 | 15.5 |
| Erode | 17.6 | 7.0 |
| Etah | 11.6 | 2.9 |
| Etawah | 11.3 | 5.2 |
| Faizabad | 7.0 | 10.9 |
| Faridabad | 20.2 | 6.5 |
| Faridkot | 25.5 | 10.4 |
| Farrukhabad | 8.0 | 2.5 |
| Fatehabad | 10.2 | 8.3 |
| Fatehgarh Sahib | 25.7 | 9.8 |
| Fatehpur | 10.4 | 2.8 |
| Fazilka | 31.2 | 8.2 |
| Firozabad | 13.2 | 4.6 |
| Firozpur | 31.7 | 7.5 |
| Gadag | 17.4 | 6.5 |
| Gadchiroli | 10.8 | 4.1 |
| Gajapati | 11.4 | 4.1 |
| Ganderbal | 17.6 | 5.0 |
| Gandhinagar | 20.1 | 11.7 |
| Ganganagar | 13.5 | 5.6 |
| Ganjam | 16.3 | 8.9 |
| Garhwa | 10.8 | 6.1 |
| Garhwal | 13.1 | 3.4 |
| Gariyaband | 16.8 | 3.4 |
| Gautam Buddha Nagar | 9.2 | 8.3 |
| Gaya | 10.8 | 4.4 |
| Ghaziabad | 12.7 | 10.1 |
| Ghazipur | 13.6 | 7.4 |
| Gir Somnath | 9.6 | 9.9 |
| Giridih | 12.9 | 6.8 |
| Goalpara | 9.8 | 6.2 |
| Godda | 9.9 | 6.2 |
| Golaghat | 12.1 | 5.3 |
| Gomati | 10.2 | 7.1 |
| Gonda | 18.5 | 4.0 |
| Gondiya | 13.1 | 3.0 |
| Gopalganj | 13.6 | 5.0 |
| Gorakhpur | 14.8 | 8.5 |
| Gulbarga | 16.5 | 8.3 |
| Gumla | 11.0 | 4.3 |
| Guna | 12.5 | 5.4 |
| Guntur | 15.9 | 13.6 |
| Gurdaspur | 33.6 | 6.5 |
| Gurgaon | 10.1 | 5.9 |
| Gwalior | 13.3 | 5.8 |
| Hailakandi | 13.2 | 7.2 |
| Hamirpur | 17.4 | 9.0 |
| Hamirpur | 16.6 | 3.2 |
| Hanumangarh | 19.3 | 4.2 |
| Haora | 17.5 | 10.9 |
| Hapur | 21.5 | 4.7 |
| Harda | 18.3 | 4.5 |
| Hardoi | 13.0 | 4.5 |
| Hardwar | 19.4 | 3.8 |
| Hassan | 21.9 | 9.0 |
| Haveri | 16.9 | 8.1 |
| Hazaribagh | 15.2 | 5.0 |
| Hingoli | 13.1 | 5.6 |
| Hisar | 15.3 | 7.1 |
| Hojai | 15.2 | 7.0 |
| Hoshangabad | 17.7 | 3.1 |
| Hoshiarpur | 37.0 | 7.1 |
| Hugli | 16.9 | 12.1 |
| Hyderabad | 20.4 | 12.5 |
| Idukki | 23.2 | 12.6 |
| Imphal East | 19.8 | 8.7 |
| Imphal West | 23.5 | 10.9 |
| Indore | 15.2 | 5.9 |
| Jabalpur | 9.8 | 8.1 |
| Jagatsinghapur | 18.2 | 9.4 |
| Jagitial | 14.5 | 7.0 |
| Jaipur | 10.6 | 3.8 |
| Jaisalmer | 8.2 | 3.5 |
| Jajapur | 16.9 | 7.8 |
| Jalandhar | 26.8 | 8.4 |
| Jalaun | 18.4 | 2.9 |
| Jalgaon | 16.8 | 7.4 |
| Jalna | 14.2 | 5.7 |
| Jalor | 7.0 | 6.8 |
| Jalpaiguri | 15.5 | 9.2 |
| Jammu | 14.1 | 3.9 |
| Jamnagar | 10.8 | 10.4 |
| Jamtara | 13.3 | 5.7 |
| Jamui | 5.9 | 4.8 |
| Jangoan | 15.3 | 7.3 |
| Janjgir - Champa | 18.5 | 4.6 |
| Jashpur | 21.9 | 3.1 |
| Jaunpur | 15.9 | 6.1 |
| Jayashankar Bhupalapally | 17.3 | 6.2 |
| Jehanabad | 5.7 | 7.6 |
| Jhabua | 14.7 | 4.0 |
| Jhajjar | 19.8 | 5.3 |
| Jhalawar | 13.1 | 3.3 |
| Jhansi | 13.2 | 3.1 |
| Jharsuguda | 13.6 | 8.5 |
| Jhunjhunun | 20.1 | 3.9 |
| Jind | 11.1 | 7.0 |
| Jodhpur | 11.9 | 4.7 |
| Jogulamba Gadwal | 14.8 | 4.8 |
| Jorhat | 16.9 | 9.7 |
| Junagadh | 16.2 | 13.2 |
| Jyotiba Phule Nagar | 18.6 | 3.3 |
| Kabeerdham | 19.6 | 4.6 |
| Kachchh | 12.9 | 6.9 |
| Kaimur (Bhabua) | 7.8 | 3.1 |
| Kaithal | 10.7 | 7.8 |
| Kalahandi | 21.2 | 7.4 |
| Kamareddy | 16.5 | 6.8 |
| Kamrup | 11.6 | 10.2 |
| Kamrup Metropolitan | 9.3 | 10.5 |
| Kancheepuram | 17.9 | 12.9 |
| Kandhamal | 16.5 | 5.5 |
| Kangra | 16.1 | 7.7 |
| Kannauj | 9.9 | 3.0 |
| Kanniyakumari | 14.6 | 14.4 |
| Kannur | 24.3 | 14.7 |
| Kanpur Dehat | 8.8 | 3.2 |
| Kanpur Nagar | 10.6 | 4.6 |
| Kanshiram Nagar | 12.3 | 3.0 |
| Kapurthala | 28.5 | 9.1 |
| Karaikal | 12.6 | 11.9 |
| Karauli | 6.3 | 2.4 |
| Karbi Anglong | 11.9 | 5.6 |
| Kargil | 14.5 | 4.7 |
| Karimganj | 11.0 | 9.0 |
| Karimnagar | 16.8 | 8.7 |
| Karnal | 16.1 | 4.3 |
| Karur | 11.9 | 11.7 |
| Kasaragod | 16.2 | 11.1 |
| Kathua | 15.7 | 6.2 |
| Katihar | 8.0 | 7.4 |
| Katni | 11.6 | 4.4 |
| Kaushambi | 3.9 | 5.7 |
| Kendrapara | 11.3 | 7.2 |
| Kendujhar | 12.2 | 5.3 |
| Khagaria | 7.3 | 10.1 |
| Khammam | 17.3 | 8.4 |
| Khandwa (East Nimar) | 11.8 | 2.7 |
| Khargone (West Nimar) | 19.7 | 5.9 |
| Kheda | 18.1 | 12.2 |
| Kheri | 15.2 | 4.4 |
| Khordha | 13.3 | 13.0 |
| Khowai | 9.6 | 8.6 |
| Khunti | 8.0 | 5.9 |
| Kinnaur | 10.0 | 1.9 |
| Kiphire | 7.2 | 2.1 |
| Kishanganj | 8.9 | 5.3 |
| Kishtwar | 6.6 | 3.3 |
| Koch Bihar | 13.1 | 7.0 |
| Kodagaon | 15.9 | 3.8 |
| Kodagu | 21.8 | 8.7 |
| Kodarma | 10.0 | 8.8 |
| Kohima | 20.5 | 2.7 |
| Kokrajhar | 11.0 | 4.8 |
| Kolar | 20.2 | 8.5 |
| Kolasib | 12.4 | 8.3 |
| Kolhapur | 28.1 | 7.8 |
| Kolkata | 14.8 | 13.8 |
| Kollam | 21.1 | 17.5 |
| Komaram Bheem Asifabad | 13.7 | 3.7 |
| Koppal | 13.2 | 5.6 |
| Koraput | 13.0 | 4.2 |
| Korba | 19.5 | 3.9 |
| Koriya | 17.6 | 6.1 |
| Kota | 14.2 | 3.2 |
| Kottayam | 25.2 | 16.9 |
| Kozhikode | 19.4 | 14.3 |
| Kra Daadi | 14.4 | 1.0 |
| Krishna | 14.0 | 12.4 |
| Krishnagiri | 13.6 | 6.1 |
| Kulgam | 16.5 | 2.2 |
| Kullu | 10.6 | 3.3 |
| Kupwara | 15.0 | 4.4 |
| Kurnool | 19.6 | 9.3 |
| Kurukshetra | 33.7 | 7.9 |
| Kurung Kumey | 15.3 | 3.6 |
| Kushinagar | 12.3 | 5.6 |
| Lahul & Spiti | 7.5 | 2.5 |
| Lakhimpur | 14.2 | 3.3 |
| Lakhisarai | 6.2 | 7.8 |
| Lakshadweep | 21.7 | 15.7 |
| Lalitpur | 15.2 | 2.5 |
| Latehar | 11.1 | 4.4 |
| Latur | 14.5 | 4.7 |
| Lawngtlai | 8.4 | 5.8 |
| Leh(Ladakh) | 10.1 | 2.9 |
| Lohardaga | 13.0 | 3.1 |
| Lohit | 15.3 | 3.7 |
| Longding | 17.2 | 7.1 |
| Longleng | 14.1 | 4.7 |
| Lower Dibang Valley | 24.3 | 3.8 |
| Lower Subansiri | 20.7 | 5.2 |
| Lucknow | 11.8 | 8.3 |
| Ludhiana | 21.7 | 7.6 |
| Lunglei | 10.1 | 8.3 |
| Madhepura | 5.8 | 8.1 |
| Madhubani | 9.4 | 6.3 |
| Madurai | 16.6 | 11.0 |
| Mahabubabad | 13.3 | 6.2 |
| Mahabubnagar | 15.9 | 4.9 |
| Mahamaya Nagar | 17.6 | 3.2 |
| Mahasamund | 13.0 | 3.2 |
| Mahe | 33.6 | 24.0 |
| Mahendragarh | 18.8 | 4.9 |
| Mahesana | 19.2 | 14.0 |
| Mahisagar | 9.8 | 7.2 |
| Mahoba | 11.8 | 3.4 |
| Mahrajganj | 16.1 | 5.9 |
| Mainpuri | 10.1 | 4.5 |
| Majuli | 15.5 | 5.3 |
| Malappuram | 17.5 | 13.6 |
| Maldah | 10.2 | 10.0 |
| Malkangiri | 9.9 | 6.5 |
| Mamit | 8.9 | 5.4 |
| Mancherial | 17.1 | 6.8 |
| Mandi | 11.5 | 7.5 |
| Mandla | 17.3 | 4.0 |
| Mandsaur | 26.1 | 4.8 |
| Mandya | 18.1 | 7.7 |
| Mansa | 31.6 | 5.6 |
| Mathura | 14.0 | 3.0 |
| Mau | 28.0 | 8.4 |
| Mayurbhanj | 16.3 | 4.1 |
| Medak | 15.9 | 7.8 |
| Medchal-Malkajgiri | 16.6 | 6.6 |
| Meerut | 16.5 | 8.2 |
| Mewat | 11.3 | 3.1 |
| Mirzapur | 17.9 | 4.5 |
| Moga | 33.4 | 7.5 |
| Mokokchung | 15.8 | 3.1 |
| Mon | 15.5 | 3.8 |
| Moradabad | 19.8 | 6.7 |
| Morbi | 14.1 | 15.8 |
| Morena | 9.1 | 7.2 |
| Morigaon | 12.4 | 5.4 |
| Muktsar | 23.7 | 8.2 |
| Mumbai | 12.1 | 9.9 |
| Mumbai Suburban | 12.5 | 9.7 |
| Mungeli | 18.1 | 4.7 |
| Munger | 9.0 | 11.8 |
| Murshidabad | 9.5 | 11.2 |
| Muzaffarnagar | 16.3 | 6.9 |
| Muzaffarpur | 11.6 | 6.5 |
| Mysore | 17.2 | 6.4 |
| Nabarangapur | 12.6 | 2.9 |
| Nadia | 9.6 | 11.3 |
| Nagaon | 9.9 | 11.2 |
| Nagapattinam | 16.1 | 9.1 |
| Nagarkurnool | 15.3 | 4.9 |
| Nagaur | 13.5 | 5.1 |
| Nagpur | 14.2 | 6.4 |
| Nainital | 18.4 | 5.6 |
| Nalanda | 7.0 | 5.2 |
| Nalbari | 13.1 | 10.1 |
| Nalgonda | 10.6 | 6.9 |
| Namakkal | 13.3 | 8.7 |
| Namsai | 19.0 | 4.2 |
| Nanded | 12.0 | 5.4 |
| Nandurbar | 19.2 | 5.6 |
| Narayanpur | 13.5 | 3.4 |
| Narmada | 14.8 | 7.0 |
| Narsimhapur | 16.0 | 6.7 |
| Nashik | 19.8 | 6.3 |
| Navsari | 17.9 | 11.2 |
| Nawada | 9.4 | 3.8 |
| Nayagarh | 12.1 | 12.4 |
| Neemuch | 22.9 | 4.0 |
| New Delhi | 16.7 | 4.4 |
| Nicobars | 30.5 | 6.5 |
| Nirmal | 13.5 | 4.9 |
| Nizamabad | 16.0 | 5.8 |
| North | 14.4 | 3.8 |
| North & Middle Andaman | 19.1 | 7.6 |
| North District | 23.8 | 3.5 |
| North East | 24.2 | 6.5 |
| North Garo Hills | 16.7 | 10.5 |
| North Goa | 19.7 | 12.3 |
| North Tripura | 11.2 | 11.5 |
| North Twenty Four Parganas | 12.0 | 13.7 |
| North West | 17.5 | 6.7 |
| Nuapada | 14.6 | 4.8 |
| Osmanabad | 19.7 | 4.8 |
| Pakur | 8.6 | 5.7 |
| Palakkad | 25.5 | 15.8 |
| Palamu | 13.7 | 4.5 |
| Palghar | 17.0 | 6.8 |
| Pali | 8.6 | 6.6 |
| Palwal | 21.0 | 5.7 |
| Panch Mahals | 21.5 | 10.4 |
| Panchkula | 15.7 | 9.5 |
| Panipat | 17.6 | 6.4 |
| Panna | 6.6 | 3.4 |
| Papum Pare | 13.1 | 2.4 |
| Parbhani | 11.2 | 4.0 |
| Paschim Barddhaman | 13.3 | 14.4 |
| Paschim Medinipur | 10.9 | 11.1 |
| Pashchim Champaran | 9.6 | 3.1 |
| Pashchimi Singhbhum | 9.6 | 2.3 |
| Patan | 9.4 | 12.3 |
| Pathanamthitta | 31.2 | 19.8 |
| Pathankot | 28.2 | 5.3 |
| Patiala | 22.2 | 9.0 |
| Patna | 8.0 | 10.6 |
| Peddapalli | 18.3 | 6.2 |
| Perambalur | 8.2 | 8.3 |
| Peren | 12.0 | 2.9 |
| Phek | 19.4 | 4.3 |
| Pilibhit | 9.1 | 5.8 |
| Pithoragarh | 18.2 | 2.9 |
| Porbandar | 9.9 | 11.6 |
| Prakasam | 15.8 | 13.2 |
| Pratapgarh | 10.4 | 3.3 |
| Pratapgarh | 12.4 | 8.8 |
| Puducherry | 18.3 | 10.4 |
| Pudukkottai | 16.6 | 10.6 |
| Pulwama | 17.8 | 2.0 |
| Punch | 12.4 | 3.4 |
| Pune | 18.3 | 6.9 |
| Purba Barddhaman | 10.7 | 10.8 |
| Purba Champaran | 9.1 | 5.9 |
| Purba Medinipur | 9.8 | 12.5 |
| Purbi Singhbhum | 15.2 | 8.4 |
| Puri | 13.2 | 13.1 |
| Purnia | 10.2 | 8.7 |
| Puruliya | 10.5 | 9.9 |
| Rae Bareli | 11.7 | 3.4 |
| Raichur | 13.3 | 5.2 |
| Raigarh | 22.4 | 4.6 |
| Raigarh | 16.9 | 5.2 |
| Raipur | 14.1 | 3.4 |
| Raisen | 12.6 | 3.8 |
| Rajanna Sircilla | 18.7 | 6.4 |
| Rajgarh | 19.7 | 3.3 |
| Rajkot | 11.1 | 10.6 |
| Rajnandgaon | 21.5 | 3.5 |
| Rajouri | 13.3 | 4.6 |
| Rajsamand | 10.3 | 2.7 |
| Ramanagara | 17.3 | 9.6 |
| Ramanathapuram | 15.0 | 9.2 |
| Ramban | 11.8 | 2.2 |
| Ramgarh | 15.4 | 4.0 |
| Rampur | 12.6 | 8.0 |
| Ranchi | 15.4 | 5.2 |
| Ranga Reddy | 22.7 | 8.6 |
| Ratlam | 20.2 | 4.1 |
| Ratnagiri | 27.7 | 9.1 |
| Rayagada | 11.4 | 5.8 |
| Reasi | 9.5 | 3.0 |
| Rewa | 9.3 | 5.6 |
| Rewari | 19.6 | 3.8 |
| Ribhoi | 8.0 | 3.4 |
| Rohtak | 17.2 | 8.3 |
| Rohtas | 9.9 | 6.1 |
| Rudraprayag | 12.3 | 4.9 |
| Rupnagar | 21.8 | 11.2 |
| Sabar Kantha | 20.4 | 9.2 |
| Sagar | 18.0 | 2.2 |
| Saharanpur | 21.1 | 5.5 |
| Saharsa | 6.0 | 5.1 |
| Sahibganj | 6.4 | 6.7 |
| Sahibzada Ajit Singh Nagar | 22.2 | 10.8 |
| Saiha | 8.9 | 3.5 |
| Salem | 16.3 | 9.4 |
| Samastipur | 6.9 | 5.5 |
| Samba | 16.4 | 6.3 |
| Sambalpur | 14.2 | 6.2 |
| Sambhal | 15.3 | 3.9 |
| Sangareddy | 16.7 | 5.2 |
| Sangli | 27.0 | 10.4 |
| Sangrur | 32.5 | 4.8 |
| Sant Kabir Nagar | 19.6 | 3.9 |
| Sant Ravidas Nagar (Bhadohi) | 12.3 | 5.6 |
| Saraikela-Kharsawan | 10.6 | 7.1 |
| Saran | 9.4 | 8.8 |
| Satara | 24.6 | 6.6 |
| Satna | 8.9 | 5.6 |
| Sawai Madhopur | 9.7 | 2.1 |
| Sehore | 21.6 | 4.3 |
| Senapati | 14.4 | 4.0 |
| Seoni | 12.7 | 5.9 |
| Sepahijala | 9.9 | 9.6 |
| Serchhip | 6.6 | 8.0 |
| Shahdara | 22.0 | 6.1 |
| Shahdol | 11.3 | 4.8 |
| Shahid Bhagat Singh Nagar | 35.5 | 10.1 |
| Shahjahanpur | 10.5 | 3.9 |
| Shajapur | 23.1 | 2.9 |
| Shamli | 21.2 | 5.4 |
| Sheikhpura | 9.3 | 4.6 |
| Sheohar | 6.4 | 2.5 |
| Sheopur | 13.8 | 4.7 |
| Shimla | 10.2 | 4.8 |
| Shimoga | 21.8 | 10.2 |
| Shivpuri | 5.9 | 4.2 |
| Shrawasti | 11.9 | 4.2 |
| Shupiyan | 9.7 | 3.7 |
| Siang | 21.6 | 3.2 |
| Siddharthnagar | 16.9 | 5.4 |
| Siddipet | 18.1 | 6.2 |
| Sidhi | 12.6 | 4.0 |
| Sikar | 17.9 | 3.8 |
| Simdega | 16.6 | 3.6 |
| Sindhudurg | 28.4 | 14.3 |
| Singrauli | 12.1 | 4.3 |
| Sirmaur | 14.7 | 4.4 |
| Sirohi | 11.3 | 1.9 |
| Sirsa | 17.7 | 13.0 |
| Sitamarhi | 7.7 | 5.3 |
| Sitapur | 11.9 | 2.4 |
| Sivaganga | 14.5 | 12.2 |
| Sivasagar | 16.4 | 8.9 |
| Siwan | 15.8 | 5.4 |
| Solan | 11.1 | 4.4 |
| Solapur | 19.9 | 5.9 |
| Sonbhadra | 13.9 | 4.8 |
| Sonipat | 15.2 | 6.9 |
| Sonitpur | 15.6 | 4.4 |
| South | 17.2 | 4.7 |
| South Andaman | 16.5 | 10.7 |
| South District | 30.7 | 5.2 |
| South East | 18.3 | 4.3 |
| South Garo Hills | 12.8 | 11.1 |
| South Goa | 18.5 | 14.6 |
| South Salmara Mancachar | 9.1 | 4.5 |
| South Tripura | 12.2 | 8.6 |
| South Twenty Four Parganas | 13.3 | 11.6 |
| South West | 15.0 | 9.0 |
| South West Garo Hills | 11.6 | 10.1 |
| South West Khasi Hills | 10.9 | 1.9 |
| Sri Potti Sriramulu Nellore | 14.3 | 12.0 |
| Srikakulam | 10.8 | 8.1 |
| Srinagar | 11.8 | 2.7 |
| Subarnapur | 14.5 | 5.7 |
| Sukma | 11.6 | 2.5 |
| Sultanpur | 14.0 | 8.2 |
| Sundargarh | 13.7 | 5.4 |
| Supaul | 4.9 | 5.4 |
| Surajpur | 19.6 | 5.5 |
| Surat | 16.2 | 9.6 |
| Surendranagar | 10.1 | 10.8 |
| Surguja | 21.7 | 4.4 |
| Suryapet | 14.3 | 10.0 |
| Tamenglong | 14.9 | 5.2 |
| Tapi | 22.2 | 11.8 |
| Tarn Taran | 29.2 | 5.4 |
| Tawang | 19.1 | 1.4 |
| Tehri Garhwal | 12.7 | 2.6 |
| Thane | 16.4 | 7.5 |
| Thanjavur | 15.4 | 11.3 |
| The Dangs | 14.3 | 2.1 |
| The Nilgiris | 22.4 | 7.4 |
| Theni | 21.6 | 13.6 |
| Thiruvallur | 17.7 | 11.9 |
| Thiruvananthapuram | 17.0 | 14.2 |
| Thiruvarur | 14.9 | 15.1 |
| Thoothukkudi | 14.2 | 13.2 |
| Thoubal | 20.3 | 4.7 |
| Thrissur | 20.6 | 16.2 |
| Tikamgarh | 4.7 | 4.9 |
| Tinsukia | 9.8 | 7.6 |
| Tirap | 15.3 | 2.8 |
| Tiruchirappalli | 11.9 | 13.7 |
| Tirunelveli | 10.9 | 10.3 |
| Tiruppur | 14.5 | 9.2 |
| Tiruvannamalai | 11.6 | 10.6 |
| Tonk | 14.2 | 2.5 |
| Tuensang | 14.9 | 2.4 |
| Tumkur | 19.8 | 8.7 |
| Udaipur | 10.5 | 4.7 |
| Udalguri | 11.6 | 7.4 |
| Udham Singh Nagar | 14.3 | 7.0 |
| Udhampur | 16.4 | 6.0 |
| Udupi | 27.6 | 11.1 |
| Ujjain | 19.3 | 3.9 |
| Ukhrul | 12.0 | 3.7 |
| Umaria | 12.9 | 5.3 |
| Una | 18.9 | 11.7 |
| Unakoti | 11.5 | 9.2 |
| Unnao | 12.5 | 3.8 |
| Upper Siang | 19.0 | 2.5 |
| Upper Subansiri | 20.6 | 3.1 |
| Uttar Bastar Kanker | 22.7 | 2.4 |
| Uttar Dinajpur | 11.1 | 10.3 |
| Uttara Kannada | 20.3 | 7.3 |
| Uttarkashi | 11.9 | 3.1 |
| Vadodara | 18.5 | 11.1 |
| Vaishali | 9.7 | 7.7 |
| Valsad | 20.8 | 10.3 |
| Varanasi | 24.4 | 8.0 |
| Vellore | 15.8 | 11.4 |
| Vidisha | 9.7 | 5.5 |
| Vikarabad | 16.8 | 5.5 |
| Viluppuram | 10.9 | 7.9 |
| Virudhunagar | 9.3 | 9.7 |
| Visakhapatnam | 14.9 | 8.6 |
| Vizianagaram | 13.7 | 5.6 |
| Wanaparthy | 13.8 | 8.0 |
| Warangal Rural | 16.5 | 7.7 |
| Warangal Urban | 18.1 | 9.7 |
| Wardha | 8.5 | 3.2 |
| Washim | 17.2 | 4.8 |
| Wayanad | 17.0 | 7.6 |
| West | 16.7 | 4.4 |
| West District | 34.1 | 5.5 |
| West Garo Hills | 14.0 | 8.4 |
| West Godavari | 17.9 | 10.1 |
| West Jaintia Hills | 8.6 | 3.1 |
| West Kameng | 16.9 | 1.8 |
| West Karbi Anglong | 10.6 | 4.2 |
| West Khasi Hills | 7.5 | 2.1 |
| West Siang | 28.7 | 5.1 |
| West Tripura | 15.5 | 8.7 |
| Wokha | 12.2 | 5.1 |
| Y.S.R. | 16.7 | 10.2 |
| Yadadri Bhuvanagiri | 18.1 | 6.8 |
| Yadgir | 13.1 | 5.6 |
| Yamunanagar | 29.0 | 9.5 |
| Yanam | 23.0 | 12.5 |
| Yavatmal | 10.5 | 4.5 |
| Zunheboto | 22.0 | 2.5 |
